# High-level prodigiosin production in *Pseudomonas putida* enabled by combinatorial metabolic engineering

**DOI:** 10.1016/j.synbio.2026.01.015

**Published:** 2026-01-30

**Authors:** Yuxin Zhang, Meiyan Wang, Kaijie Dou, Ruizhi Zhang, Chunfang Wang, Xiaoying Bian, Jun Si, Guoqing Niu

**Affiliations:** a College of Agronomy and Biotechnology, Southwest University, Chongqing, 400715, China; b Institute of Biotechnology, Shanxi University, Taiyuan, 030006, Shanxi, China; c College of Pharmaceutical Sciences and Chinese Medicine, Southwest University, Chongqing, 400715, China; d Southwest University Hospital, Chongqing, 400715, China; e Helmholtz International Lab for Anti-infectives, Shandong University-Helmholtz Institute of Biotechnology, State Key Laboratory of Microbial Technology, Shandong University, Qingdao, Shandong, 266237, China; f College of Horticulture and Landscape Architecture, Southwest University, Chongqing 400715, China

**Keywords:** Heterologous expression, *Pseudomonas putida*, Metabolic engineering, Prodigiosin

## Abstract

Prodigiosin, a bioactive tripyrrole pigment, exhibits a broad spectrum of biological activities-including antimicrobial, anticancer, and antimalarial properties-thereby holding significant promise for use in pharmaceutical applications and industrial biotechnology. In this study, three isolates of *Serratia marcescens* were recovered from the cabbage rhizosphere. Genomic analysis revealed a highly conserved prodigiosin biosynthetic gene cluster embedded within the chromosomes of all three isolates. Though prodigiosin production was detected in these three *S. marcescens* isolates, the relatively low yield severely limits the feasibility of its large-scale production. To address this issue, we employed a stepwise strategy involving heterologous expression, promoter engineering, genome-wide transposon mutagenesis, and optimization of fermentation media with the aim to achieve high-level prodigiosin production. The introduction of an engineered prodigiosin gene cluster into a tailored *Pseudomonas putida* KT2440 chassis strain yielded a maximum prodigiosin titer of 665 mg/L in shake-flask cultures, significantly outperforming production levels of the native *S*. *marcescens* isolates. When cultured in a small-scale stirred-tank bioreactor, the engineered strain further elevated the prodigiosin yield to 1161 mg/L. Our study presents a robust platform for prodigiosin overproduction, which can be adapted to improve the titers of other prodiginine family compounds.

## 1 Introduction

Prodigiosin is a red tripyrrole pigment belonging to the prodiginine family [1,2]. Prodiginines can be classified into linear and cyclic types based on their structural characteristics. Representative linear derivatives include prodigiosin and undecylprodigiosin, whereas metacycloprodigiosin, cycloprodigiosin and streptorubin B fall under the category of cyclic derivatives [3,4]. As a typical secondary metabolite, prodigiosin is predominantly produced by *Serratia marcescens* strains. Other bacterial species, such as *Hahella chejuensis*, *Pseudoalteromonas rubra*, *Vibrio psychroerythrous* and *Zooshikella rubidus*, also serve as sources of prodigiosin [3,[5], [6], [7]]. Prodigiosin exhibits excellent biological activities, such as antimicrobial, anticancer, algicidal, antimalarial, and antiprotozoal properties, and therefore it holds significant promise for applications in cosmetics, pharmaceutical, biomedical, and food industries [[8], [9], [10], [11], [12]].

The gene cluster responsible for prodigiosin biosynthesis was first identified in *S. marcescens* ATCC 274 [13]. The *pig* gene cluster consists of 14 genes spanning a contiguous DNA region of approximately 21 kb, arranged sequentially from *pigA* to *pigN*. Among these genes, *pigB*, *pigD* and *pigE* encode biosynthetic enzymes that catalyze the formation of 2-methyl-3-pentylpyrrole (MAP) from the precursors 2-octenal and pyruvate [14,15]. PigA and PigF-N are proposed to catalyze the biosynthesis of 4-methoxy-2,2′-bipyrrole-5-carbaldehyde (MBC) using proline and malonyl-CoA as substrates. However, the specific role of each enzyme remains to be fully elucidated [1,16,17]. Finally, PigC catalyzes the condensation of MAP and MBC to yield the final product prodigiosin. The completely distinct biosynthetic pathways for MAP lead to structural variations of compounds among prodiginine-producing microorganisms, offering significant potential for the development of novel prodiginine derivatives [18].

Continuous strain improvement is essential for optimizing the fermentation-based manufacturing of high-value natural products. Metabolic engineering has emerged as an increasingly powerful approach for enhancing the titers of natural products, encompassing strategies such as reconstituting entire pathways in heterologous hosts, boosting precursor supply, and fortifying the regulation of gene expression [[19], [20], [21]]. In previous studies, heterologous expression of the *pig* gene cluster was successfully established in *Escherichia coli* and *Erwinia carotovora*; however, quantitative prodigiosin yields were not reported in these works [22,23]. In our recent work, a *pig* gene cluster was captured from *S. marcescens* BoR121, an isolate of the cabbage rhizosphere. Heterologous expression of the gene cluster in *E. coli* BAP1 resulted in prodigiosin production with a yield of 101 μg/g [24]. Several studies have demonstrated that *Pseudomonas putida* KT2440 represents a preferred surrogate host for prodigiosin production. For example, the *pig* gene cluster from *S. marcescens* was integrated into *P. putida* KT2440 *via* transposon-mediated gene transfer, yielding prodigiosin production titers of 25–150 mg/L [[25], [26], [27]]. In another study, *P. putida* KT2440 was engineered by modulating the synthesis of outer membrane vesicles (OMVs), which led to a threefold increase in prodigiosin production-with the final titer reaching approximately 60–70 mg/L [28]. In a recent study, the same gene cluster was integrated into *P. putida* KT2440 by using Cas9-assisted homologous recombination. When the gene cluster was placed under the control of the rhamnose-inducible *rha* promoter, the resulting strain (PIG01) achieved a prodigiosin titer of approximately 500 mg/L. Furthermore, when the gene cluster was placed under the control of the IPTG-inducible *trc* promoter, the resulting strain (PIG02) reached a prodigiosin titer of approximately 1.1 g/L [29].

In this study, three *S. marcescens* isolates were obtained from the rhizosphere of cabbage (*Brassica oleracea* var. *capitata*), and genomic analysis revealed that all three isolates harbored a conserved gene cluster for prodigiosin biosynthesis. High-performance liquid chromatography (HPLC) and liquid chromatography-mass spectrometry (LC-MS) analyses further confirmed the production of prodigiosin in all three strains. However, the prodigiosin yield was relatively low, with titers ranging from 1.6 to 1.7 mg/L. To address this issue, we developed a rational, systematic approach to enhance prodigiosin titer in the surrogate host *P. putida* KT2440, achieving stepwise improvements in production through the following strategies. First, replacing the native promoter of the *pig* gene cluster with the constitutive *P*_*46*_ promoter increased prodigiosin production to 512 mg/L, a significant improvement over the native producers. Second, engineering the gene cluster to incorporate an artificial malonyl-CoA biosynthesis pathway raised the titer to 601 mg/L. Third, Tn5 transposon mutagenesis of *P. putida* KT2440 yielded a prodigiosin titer of 587 mg/L. Fourth, optimizing fermentation media further boosted the titer to 665 mg/L in shake-flask cultures. Finally, scaling up cultivation of the engineered strain in a 5 L stirred-tank bioreactor achieved a prodigiosin yield of 1161 mg/L. This stepwise strategy may have broad applicability for boosting the production of high-value natural products in industrial microbial chassis strains.

## 2 Materials and methods

### 2.1 Bacterial strains, plasmids, primers, and culture conditions

Bacterial strains, plasmids, and primers used in this study are listed in Tables S1, S2, and S3 of the Supplementary Material, respectively. *P. putida* strain KT2440 served as the surrogate host for prodigiosin production. *E. coli* DH5α was used as a general host for propagating plasmids. *E. coli* BW25113 (pIJ790) was used for the construction of recombinant plasmids *via* λ-Red-mediated recombination technology [30]. *E. coli* S17-1 was used as a host for transferring DNA from *E. coli* to *Pseudomonas via* conjugation [31]. *E. coli* and *P. putida* KT2440 were cultivated in Luria-Bertani medium (LB) at 37 °C and 30 °C, respectively. *S. marcescens* strains were cultured in LB. For prodigiosin production, the engineered *Pseudomonas* strains were cultured in RK medium [29]. The medium contained 13.3 g/L KH_2_PO_4_, 4.0 g/L (NH_4_)_3_PO_4_, 1.7 g/L citric acid, 0.1 g/L ammonium ferric citrate, and 25 g/L glycerol. It was supplemented with 10 mL/L of a 100 × RK trace mineral solution and 10 mL/L of 120 g/L MgSO_4_⋅7H_2_O. The pH was adjusted to 6.7 using 5 M KOH. The 100 × RK trace mineral solution was formulated as follows (per 500 mL final volume): 0.42 g EDTA, 0.125 g CoCl_2_·6H_2_O, 0.75 g MnCl_2_·4H_2_O, 0.06 g CuCl_2_, 0.15 g H_3_BO_3_, 0.125 g Na_2_MoO_4_·2H_2_O, and 0.65 g Zn(CH_3_COO)_2_·2H_2_O. When necessary, antibiotics were used at the following concentrations: chloramphenicol (25 μg/mL for *E. coli*), gentamycin (50 μg/mL for *E. coli* and *P. putida* KT2440), kanamycin (50 μg/mL for *E. coli*, 150 μg/mL for *P. putida* KT2440), and spectinomycin (150 μg/mL for *P. putida* KT2440).

### 2.2 Plasmid construction

The *pig* gene cluster was cloned from genomic DNA of BoR121 through Red/ET recombineering, leading to the generation of p15A:*pig* [24]. For the construction of pSEVA:*pig*UpDn, the upstream and downstream fragments of the gene cluster were amplified from the genomic DNA of *S. marcescens* BoR121 using primer pairs pig UpF/R and pig DnF/R, respectively. The upstream fragment was digested with *Eco*RI/*Xho*I, and the downstream fragment was digested with *Xho*I/*Hin*dIII. The two fragments were then ligated into *Eco*RI/*Hin*dIII double-digested pSEVA-gRic6T in a three-piece ligation reaction, resulting in the generation of pSEVA:*pig*UpDn. The gene cluster was transferred into pSEVA-gRic6T *via* λ-Red-mediated recombination with *Xho*I linearized pSEVA:*pig*UpDn to obtain pSEVA:*pig*. To engineer the gene cluster, a total of eight promoters were selected to evaluate their performance in *P. putida* KT2440. Four promoters, *P*_*35*_, *P*_*46*_, LvaR/*P*_*lvaA*_, and *P*_*Rox3061*_, were amplified from the genomic DNA of *P. putida* KT2440 with primers listed in Table S3. Three promoters, *P*_*trc*_, BG42, and *P*_*rpsJ12*_, were synthesized by GenScript (GenScript, Nanjing, Jiangsu, China). The *P*_*cgtrc*_ promoter was amplified from pTrcmob with primer pair cgtrc pF/R [32]. Similar to the construction of pSEVA:*pig*, the recombinant plasmids pSEVA::*P*_*trc*_-*pig*, pSEVA::*P*_*cgtrc*_-*pig*, pSEVA::BG42-*pig*, pSEVA::*P*_*rpsJ12*_-*pig*, pSEVA::*P*_*35*_-*pig*, pSEVA::*P*_*46*_-*pig*, pSEVA::LvaR/*P*_*lvaA*_-*pig*, and pSEVA::*P*_*Rox3061*_-*pig*, were generated using λ-Red-mediated recombination.

To incorporate the artificial malonyl-CoA pathway, *bauA* and *mcrC* genes were amplified with pCDF-*bauA*-*mcrC* as template by using primers bauA F and mcrC R [33]. The amplicon was inserted into pSEVA::*P*_*46*_-*pig*UpDn *via* homologous recombination technology following the manufacturer's instructions (ClonExpress MultiS One Step Cloning Kit, Vazyme Biotech Co., Ltd.). Next, the original T7 promoter was replaced with three alternative promoters to drive the expression of *bauA* and *mcrC*. The resulting plasmids were linearized with *Xho*I and then used to obtain pSEVA::*P*_*46*_-*pig*-*P*_*46*_-*bauA*-*mcrC*, pSEVA::*P*_*46*_-*pig*-*P*_*Rox3061*_-*bauA*-*mcrC*, and pSEVA::*P*_*46*_-*pig*-*P*_*rha*_-*bauA*-*mcrC via* λ-Red-mediated recombination.

### 2.3 Intergeneric conjugation

Intergeneric conjugation between *E. coli* and *Pseudomonas* was performed essentially as described previously [31]. Briefly, *E. coli* S17-1 containing the conjugative plasmid and *P. putida* KT2440 were grown in LB until the optical density at 600 nm (OD_600_) reached 0.6. All cells were concentrated and washed four times with double-distilled water, then *E. coli* was mixed with *Pseudomonas* in a ratio of 1:1. These mixtures were dripped on LB agar plates in small dots, and incubated at 37 °C for 6 h, then shifted to 30 °C for 16–18 h. After incubation, cells were scraped from the plate using 1 mL double-distilled water, serially diluted, and plated on LB selection plates containing gentamicin (50 μg/mL) and spectinomycin (150 μg/mL). Plates were incubated at 30 °C for an additional 2–3 days.

### 2.4 Production and analysis of prodigiosin

For prodigiosin production, strains were cultivated in LB at 30 °C on a rotary shaker (220 rpm) overnight as a seed culture. The optical density of the seed culture suspension was measured at 650 nm and then normalized to a value of 1.0. A 2 % inoculum of seed culture was transferred into a shake-flask containing 25 mL fermentation medium. The cultures were cultivated at 30 °C for 72 h. When necessary, l-rhamnose, pyruvate, proline or 2-octenal was added to the fermentation medium until OD_650_ reached 0.5.

For prodigiosin analysis, 1 mL of culture was then centrifuged at 13,000 g for 10 min to discard the supernatant. The pellet was resuspended in 1 mL of acidified methanol (4 % (v/v) 1 M HCl in methanol) and sonicated for 20 min. This extraction procedure was repeated twice, and the supernatants were filtered through a Millipore membrane (pore diameter, 0.22 μm). HPLC analysis was performed with an SHIMADZU LC-20A HPLC system and a ZORBAX SB-C18 column (5 μm pore size; 4.6 mm × 250 mm). LC-MS analysis was performed on Agilent 1290 Infinity LC System/6230. LC/MS system with a Rapid Resolution HD C18 column (1.8 μm pore size; 2.1 × 150 mm, Agilent, ZORBAX Eclipse Plus). HPLC conditions for prodigiosin analysis were implemented as described previously [24], with the detailed parameters specified as follows. The mobile phase consisted of solvent A (0.1 % formic acid) and solvent B (methanol). The flow rate was maintained at 1 mL/min, and UV detection was conducted at a wavelength of 535 nm. The elution gradient program was set as follows: 0–2 min, linear gradient elution with solvent B increasing from 50 to 100 %; 2–5 min, isocratic elution with 100 % solvent B; 5–7min, linear gradient elution with solvent B decreasing from 100 to 50 %; and 7–20 min, isocratic elution with 50 % solvent B. Quantification of prodigiosin production was achieved by using a standard curve generated with authentic standard. The linear regression equation for this curve was: y = 346576x − 561106. In this equation, y represents the measured HPLC peak area of prodigiosin, x denotes the prodigiosin concentration (in mg/L). The coefficient of determination (R^2^ = 0.9917) indicated a strong linear correlation between peak area and concentration, thereby validating the calibration curve for accurate quantitative analysis.

### 2.5 Mutagenesis library construction

The EZ-Tn5™ <KAN-2>Tnp Transposome™ Kit (Lucigen) was used for the construction of mutagenesis library following the manufacturer's instructions. The parent strain was cultured in 10 ml LB medium supplemented with the appropriate antibiotic at 30 °C. When the OD_600_ value of the culture reached approximately 0.6–0.8, the culture was placed in an ice-water mixture and incubated for 10 min. The cells were harvested by centrifugation at 5000*g* for 5 min to remove the supernatant. Then, the cells were washed three times with 10 % (v/v) glycerol and resuspended in a final volume of 500 μL of 10 % (v/v) glycerol. Next, 1 μL EZ-Tn5 transposon was introduced into 100 μL bacterial suspension by electroporation. Next, 1 mL LB liquid medium was added and incubated at 30 °C and 220 rpm for 2 h. The incubated broth was diluted properly and screened on LB agar plates containing kanamycin (50 μg/mL) and gentamycin (50 μg/mL). Plates were incubated at 30 °C until the colonies turned red. Candidate colonies were fermented in 48-deep-well plates with RK liquid media, and treated with 1 mL of acidified methanol after fermentation. The OD_535_ of the supernatants was assayed using the multimode Varioskan LUX microplate reader (Thermo Scientific). Strains exhibiting higher absorption values than the parent strain were cultured in shaking flasks containing RK medium to further quantify prodigiosin production. The transposon insertion site for each mutant was determined using arbitrary priming PCR as described previously [34,35].

### 2.6 Construction of deletion mutant strains and genetic complementation

All mutant strains were obtained *via* CRISPR/Cas9-mediated genome editing. The system consists of two plasmids, the *cas9* gene and the sgRNA that direct it to the targeted region are separated in the pCas-RK2K and pSEVA-gRic6T series [31]. Plasmid pCAS-RK2K was first transformed into *P. putida* KT2440 by E*. coli*-*Pseudomonas* intergeneric conjugation. For the construction of pSEVA::D1780 UpDn, two fragments, covering appropriately 500 bp of homologous upstream and downstream of *PP*_1780, were amplified by PCR from the genomic DNA of *P. putida* KT2440 using primer pairs D1780 Up F/R and D1780 Dn F/R. The two fragments were then assembled together by using overlap extension PCR with primer pair with D1780 Up F/D1780 Dn R. The resulting amplicon was digested with *Bam*HI/*Hin*dIII and then inserted into the corresponding sites of pSEVA-gRic6T. A similar strategy was used for the construction of pSEVA::D4187 UpDn, pSEVA::D5245 UpDn, pSEVA::D2148 UpDn, pSEVA::D2080 UpDn, pSEVA::D2126 UpDn, and pSEVA::D1182 UpDn. After confirmation by restriction digestion analysis, these plasmids were transferred into *P. putida* KT2440 harboring pCAS-RK2K by electroporation as described previously [31]. Candidate mutants were verified by PCR amplifications with primers listed in Table S3 of the Supplementary Material. For genetic complementation assays, a DNA fragment containing *PP_4187* coding sequence and its upstream promoter region was amplified from the genomic DNA of *P*. *putida* KT2440 using primer pair C4187 F/R. This amplicon was inserted into *Hin*dIII-linearized pSEVA::*P*_*46*_-*pig via* homologous recombination. The resulting pSEVA::*P*_*46*_-*pig*-C4187 was then transformed into the *PP_4187* deletion mutant, yielding the corresponding complemented strain.

### 2.7 Cultivations in stirred-tank bioreactor

Batch experiments were performed in a 5 L stirred-tank bioreactor (Biotech-5BG-7000, China) equipped with pH, temperature and dissolved oxygen probes. Seed cultures were prepared in LB media. The cells were then harvested, washed, and resuspended in RK liquid medium before a 10 % (v/v) inoculum was transferred to a bioreactor containing 2.3 L of RK fermentation. The dissolved oxygen concentration (DO) was maintained above 35 % air saturation by automatically increasing the stirrer speed up to 600 rpm and the aeration up to 4.0 vvm (air/culture volume/min). Ammonia solution (25 %, v/v) and citric acid solution (10 %, w/v) were added to adjust and maintain the pH at approximately 6.7. The culture was fermented at 30 °C for 96 h. A total of 180 mL feeding solution containing 50 % (w/v) glycerol, with MgSO_4_ at 4.5 % (w/w) of the glycerol, was supplemented into the fermenter from 36 h to 72 h of fermentation. Samples were taken every 12 h to measure prodigiosin concentration, optical density at OD_650_, and residual glycerol. Glycerol in the fermentation broth was measured using the Copper Glycerol method as previously described [36]. To determine the dry cell weight (DCW), 10 mL of fermentation broth was subjected to centrifugation at 13,000*g* for 10 min to harvest the cellular biomass. The collected cells were rinsed three times with deionized water to remove residual medium components, and subsequently dried to a constant weight at 65 °C for 12 h prior to gravimetric quantification of cell mass.

### 2.8 Data analyses

All data are average values of independent experiments calculated with their standard deviations by using GraphPad Prism 8. Statistical analyses involving multiple comparisons were performed with one-way ANOVA by IBM SPSS Statistics 21. Genome sequencing of *S. marcescens* BoR121 was conducted by Shanghai Majorbio Bio-pharm Technology Co., Ltd. using PacBio RS II and Illumina HiSeq platforms. Genome sequencing of *S. marcescens* BoR4-1 and BoR4-11 was conducted by Biomarker Technologies Corporation for the construction of Illumina short-read libraries that were subsequently sequenced on the PacBio Sequel and Illumina Hiseq platforms. The antiSMASH 6.0 was used for the detection of gene clusters encoding secondary metabolites [37]. Easyfig was employed to visualize sequence alignments among the prodigiosin gene clusters of three *S. marcescens* isolates [38].

## 3 Results

### 3.1 Identification of two additional *S*. *marcescens* isolates capable of producing prodigiosin

**Fig. 1:**
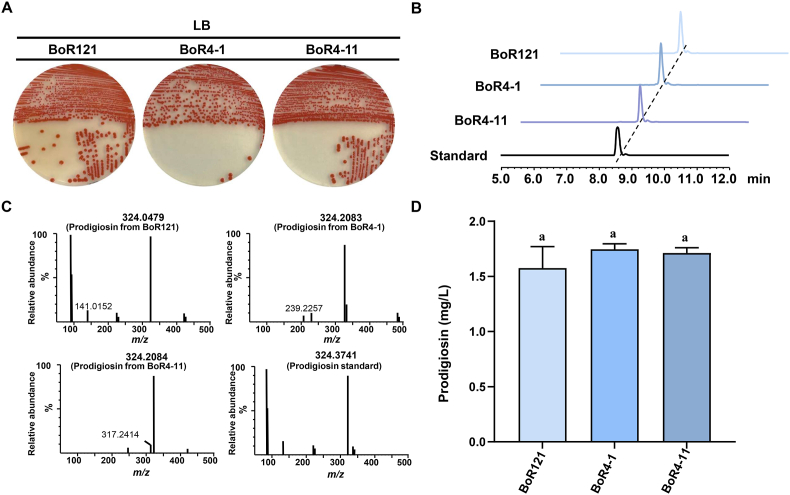
Identification of prodigiosin production in three *S. marcescens* isolates. (A) Images of three *S. marcescens* isolates cultured on LB agar medium for 2 days. (B) HPLC analysis of extracts from BoR121, BoR4-1, BoR4-11, and the prodigiosin standard. (C) LC-MS analysis of prodigiosin from BoR121, BoR4-1, BoR4-11, and the prodigiosin standard. (D) Prodigiosin yield of three *S. marcescens* isolates cultivated in LB liquid media. Error bars represent standard deviations. Mean values with the same superscript letters are not significantly different.

In our previous study, BoR121, a strain of *S. marcescens* capable of producing prodigiosin, was isolated from the cabbage rhizosphere [24]. Subsequent isolation efforts yielded two additional strains, BoR4-1 and BoR4-11. When grown on LB agar plates, both BoR4-1 and BoR4-11 displayed a distinct bright red pigmentation similar to that of BoR121 (Fig. 1A). Through 16S rRNA gene sequencing, both BoR4-1 and BoR4-11 were also identified as *S. marcescens*. To examine prodigiosin production, both BoR4-1 and BoR4-11 were cultivated in LB medium, and their culture filtrates were then subjected to HPLC analysis. Consistent with the profile of BoR121, a distinctive peak corresponding to prodigiosin was detected in the culture filtrates of BoR4-1 and BoR4-11 (Fig. 1B). To further confirm that the identity of this compound, LC-MS analysis was performed in a positive ion mode. The analysis revealed a distinct mass that matches exactly to that of authentic standard (Fig. 1C). Next, the three strains were cultured in LB medium and the extracts were subjected to HPLC analysis for prodigiosin quantification. The results showed that yields of prodigiosin in BoR121, BoR4-1, and BoR4-11 were approximately at 1.6 mg/L, 1.7 mg/L, and 1.7 mg/L, respectively (Fig. 1D). In conclusion, three *S. marcescens* strains capable of producing prodigiosin were successfully isolated from the cabbage rhizosphere. However, the yields of prodigiosin in the three native producers were quite low.

### 3.2 Genomic analysis unveils the presence of a conserved gene cluster for prodigiosin biosynthesis

**Fig. 2:**
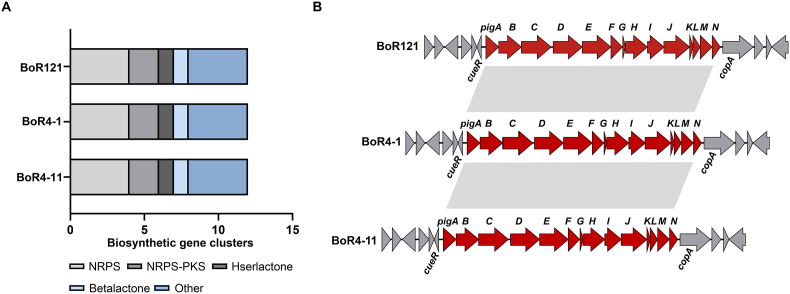
Genome analysis of three *S*. *marcescens* strains. (A) Putative BGCs content in *S. marcescens* strains as identified by the antiSMASH analysis. A total of 12 gene clusters encoding secondary metabolites were identified within the genome sequences of the three isolates. NRPS: non-ribosomal peptide synthetase; NRPS/PKS: non-ribosomal peptide synthetase-polyketide synthetase. (B) Comparative analysis of the prodigiosin gene cluster in three *S. marcescens* strains. Genetic organization of the gene cluster was highly conserved in the three isolates.

Since genome sequence of BoR121 has been acquired in our previous investigation [24], next-generation sequencing was carried out to obtain complete genome sequences of BoR4-1 and BoR4-11. With all sequences available, antiSMASH was used for the detection of secondary metabolite biosynthetic gene clusters (smBGCs) within the genome sequences of the three isolates. The analysis revealed an identical repertoire of 12 smBGCs across all three strains. These gene clusters were classified into distinct categories according to their key biosynthetic enzymes or predicted products. Specifically, including 4 clusters encoded by non-ribosomal peptide synthetases (NRPS), 2 clusters encoded by non-ribosomal peptide synthetase-polyketide synthetase (NRPS-PKS), 1 betalactones cluster, 1 hserlactones cluster, and 4 clusters belonging to other types (Fig. 2A). Further analysis revealed three gene clusters that are highly homologous to known BGCs encoding prodigiosin, yersinopine, and rhizomide A [39,40]. The remaining 9 gene clusters exhibited low similarity to BGCs that encode known compounds, indicating their potential to produce novel secondary metabolites. In BoR121, the *pig* gene cluster spanned approximately 21 kb and comprised 14 key genes crucial for prodigiosin biosynthesis. Similarly, genetic organization of the gene cluster was highly conserved in BoR4-1 and BoR4-11 (Fig. 2B). It is worth noting that the *pig* gene cluster in BoR4-1 and BoR4-11 shares sequence similarity of 100 % at the nucleotide level to that of BoR121. This genomic evidence not only validates the phenotypic observation of prodigiosin production, but also provides a foundation for elucidating the evolutionary history and potential horizontal transfer of the gene cluster among *S. marcescens* strains.

### 3.3 Engineering of the gene cluster to improve prodigiosin production

**Fig. 3:**
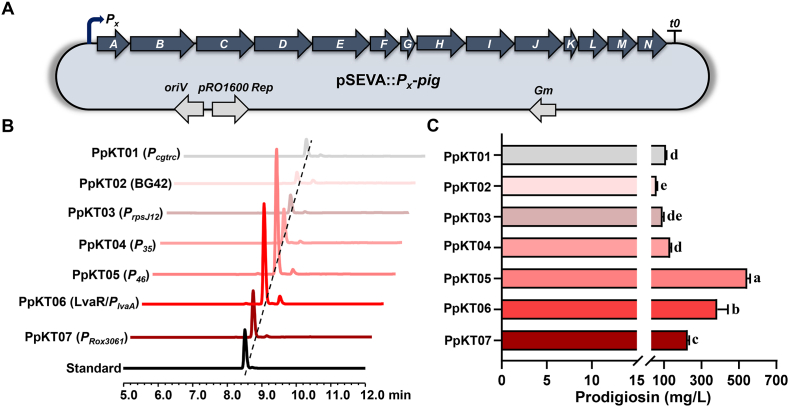
Assessment of promoters and engineering of the prodigiosin gene cluster. (A) Genetic organization of the prodigiosin gene cluster. The native promoter of *pigA* was replaced with seven alternative promoters. The arrow indicates the direction of transcription. (B) HPLC analysis of extracts from PpKT01-07, and the prodigiosin standard. Promoters used to drive the expression of the gene cluster are indicated in the bracket. All strains were cultivated in RK medium. (C) Prodigiosin yield in fermentation broths from PpKT01-07. Error bars represent standard deviations. Mean values with the same superscript letters are not significantly different, while those with different superscript letters are significantly different.

Genes responsible for prodigiosin biosynthesis are typically organized into an operon comprising 14 open reading frames (ORFs), spanning from *pigA* to *pigN* (Fig. 3A). Notably, no cluster-situated regulators were detected within this gene cluster. As mentioned earlier, *P. putida* KT2440 has emerged as a preferred surrogate host for prodigiosin production [25,29]. To enable prodigiosin production in *P. putida* KT2440, the entire *pig* gene cluster was transferred into pSEVA through λ-Red-mediated recombination (Fig. S1). The resulting recombinant plasmid, pSEVA::*pig*, was subsequently introduced into *P. putida* KT2440. The prodigiosin titer of the engineered strain reached approximately 1.7 mg/L (Fig. S2), which is comparable to that of native producers. Considering this, we decided to employ promoter engineering to enhance prodigiosin productivity in *P. putida* KT2440. As an initial approach, we utilized the *trc* promoter to drive the expression of the *pig* gene cluster. This modification resulted in a modest increase in prodigiosin titer, with the engineered strain achieving a yield of approximately 2.6 mg/L (Fig. S2).

To address this issue, our attention was dedicated to seven other well-characterized promoters. Among these, aside from the constitutive *trc* promoter derived from *Corynebacterium glutamicum* (designated *P*_*cgtrc*_), four promoters, namely BG42, *P*_*rpsJ12*_, *P*_*35*_, and *P*_*46*_, were identified as constitutive promoters in *P. putida* [[41], [42], [43]]. Additionally, the LvaR/*P*_*lvaA*_ inducible expression systems have been functionally validated in *P. putida* KT2440, where gene expression can be efficiently triggered by levulinic acid (LA) [44]. Meanwhile, *P*_*Rox3061*_ was identified as a cell density-dependent inducible promoter in *P. putida* KT2440 [45]. Each of these promoters was individually inserted upstream of *pigA* to drive the expression of the gene cluster (Fig. 3A). The resulting seven constructs were then transferred into *P. putida* KT2440, generating PpKT01-PpKT07, respectively (Fig. 3B). Analysis of prodigiosin production in these recombinant strains revealed a significant increase in all cases where the gene clusters were engineered. Among them, the gene cluster driven by *P*_*46*_ exhibited the most significant boost in prodigiosin production, followed by those driven by LvaR/*P*_*lvaA*_ and *P*_*Rox3061*_. Significantly, the titers of prodigiosin reached 512 mg/L in PpKT05, 383 mg/L in PpKT06 and 226 mg/L in PpKT07 (Fig. 3C). These results clearly demonstrate that overexpression of the *pig* gene cluster in *P. putida* KT2440 led to a substantial increase in prodigiosin titer, highlighting the effectiveness of our promoter engineering approach.

### 3.4 Incorporation of an artificial pathway for malonyl-CoA biosynthesis to improve prodigiosin production

**Fig. 4:**
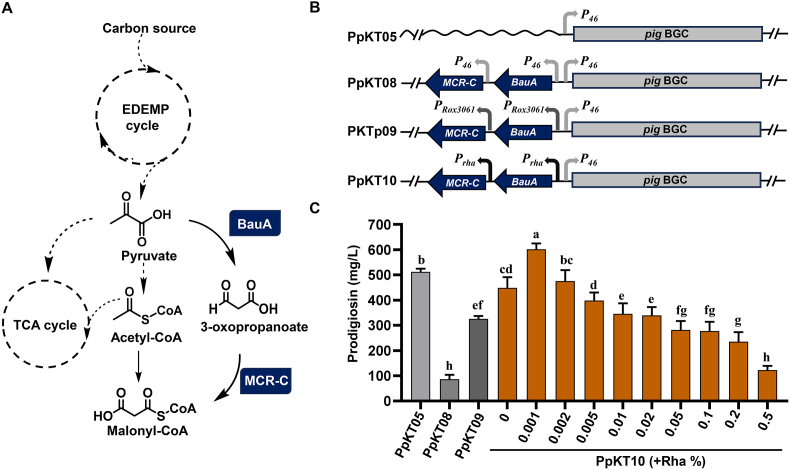
Effect of the incorporation of malonyl-CoA biosynthesis pathway on prodigiosin production. (A) A simplified schematic diagram showing the artificial pathway for malonyl-CoA biosynthesis. (B) A schematic diagram showing constitutive and inducible modules for malonyl-CoA expression. The *P*_*46*_ promoter-driven *pig* gene cluster was included to serve as a control. (C) Prodigiosin yield in fermentation broths from strains as indicated. For PpKT10, the strain was cultivated in RK medium supplemented with increasing concentrations of rhamnose. Error bars represent standard deviations. Mean values with the same superscript letters are not significantly different, while those with different superscript letters are significantly different.

In a previous investigation, an artificial pathway for malonyl-CoA biosynthesis was devised to enhance the production of malonyl-CoA-derived products (MDPs) [33]. This pathway comprises β-alanine-pyruvate transaminase (BauA) and C-terminal of a malonyl-CoA reductase (MCR-C) (Fig. 4A). To enable its application in *P. putida* KT2440, the artificial pathway was engineered with three different promoters (*P*_*46*_, *P*_*Rox3061*_, and *P*_*rha*_) and assembled with the *P*_*46*_-driven *pig* gene cluster to generate pSEVA::*P*_*46*_-*pig*-*P*_*46*_-*bauA*-*mcrC*, pSEVA::*P*_*46*_-*pig*-*P*_*Rox3061*_-*bauA*-*mcrC*, and pSEVA::*P*_*46*_-*pig*-*P*_*rha*_-*bauA*-*mcrC* (Fig. 4B). Given the fact that the same promoter was individually inserted upstream *bauA* and *mcrC*, an identical strategy was employed for our constructs. Introduction of these recombinant plasmids into *P. putida* KT2440 resulted in the generation of PpKT08, PpKT09, and PpKT10, respectively. Prodigiosin production in PpKT08 was substantially lower than that in PpKT05, suggesting that constitutive expression of the malonyl-CoA pathway has a detrimental impact on prodigiosin production. A similar observation was noted with PpKT09, in which the malonyl-CoA pathway was driven by the cell density‐dependent auto-inducible promoter *Rox3061* (Fig. 4C). Notably, upon induction with 0.001 % rhamnose, PpKT10 demonstrated a significant increase in prodigiosin production compared to PpKT05, reaching a yield of 601 mg/L. These results strongly indicate that proper modulation of the malonyl-CoA pathway is pivotal for achieving an increase in prodigiosin production.

### 3.5 Construction of a Tn5-tagged mutant library to unlock prodigiosin-producing potential

To unlock the prodigiosin-producing potential of *P. putida* KT2440, our focus was redirected to identifying genes that might exert a negative impact on prodigiosin production. In a previous study, a Tn5 transposon system was applied to identify genes influencing the levels of production of undecylprodigiosin (RED) in *S. coelicolor* [46]. Based on findings from this work, we initially selected five genes in which transposon insertions were found to significantly enhance RED production. Their orthologs in *P. putida* KT2440 were then identified *via* sequence alignment. Each of these five genes was individually deleted *via* CRISPR/Cas9-mediated genome editing. Unfortunately, prodigiosin production in these mutant strains was significantly lower than that of PpKT05 (Fig. S3), suggesting that deletion of these genes exerts an adverse effect on prodigiosin biosynthesis.

**Fig. 5:**
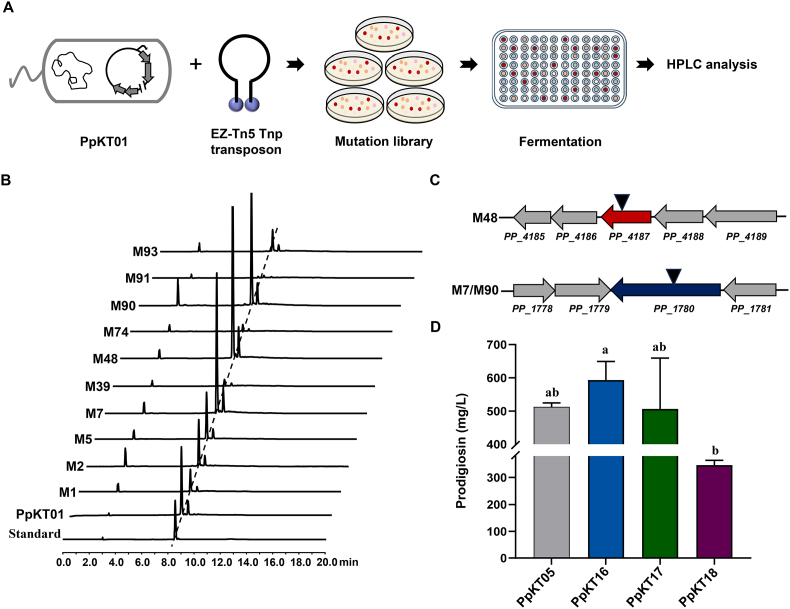
Genome-wide transposon mutagenesis to identify targets that negatively affect prodigiosin biosynthesis. (A) A simplified schematic diagram showing the construction of a mutant library in engineered *P. putida* KT2440 by Tn5-mediated transposon mutagenesis. (B) HPLC analysis of prodigiosin production in extracts from selected mutant strains. (C) Verification of the Tn5 transposon insertion sites in mutant strains with improved prodigiosin yield. (D) Prodigiosin production in gene inactivated mutant strains as indicated. Error bars represent standard deviations. Mean values with the same superscript letters are not significantly different, while those with different superscript letters are significantly different.

Our attention was then redirected to constructing a genome-wide single-gene knockout library in *P. putida* KT2440. To this end, EZ-Tn5 transposon-mediated mutagenesis was employed to generate a random mutant library using PpKT01 as the parent strain. From the 1100 obtained strains, we selected 200 strains that displayed distinct red pigmentation for screening in 48-deep-well plates. Among these, extracts from ten mutants exhibited a higher relative absorbance at 535 nm than that from the parental strain. Ultimately, these ten mutant strains were chosen for shake-flask cultures and subsequent HPLC analysis (Fig. 5A and B). The results revealed that a substantial increase in prodigiosin production was detected in three mutants, M7, M48, and M90. The precise insertion sites of the transposon were then identified *via* thermal asymmetric interlaced PCR [35]. The results revealed that the M7 and M90 mutant strains shared identical insertion sites within the *PP*_*1780* gene, whereas the M48 strain harbored an insertion in *PP*_*4187* (Fig. 5C). Sequence analysis indicated that *PP*_*4187* encodes a dihydrolipoyl dehydrogenase, a cofactor-binding subunit of both pyruvate and 2-ketoglutarate dehydrogenase complexes [47]. *PP*_*1780* encodes a putative mannosyltransferase, which is a type of glycosyltransferase that participates in lipopolysaccharide synthesis within the microbial outer membrane [48]. To further verify the involvement of these two genes in prodigiosin production, *PP*_*4187* and *PP*_*1780* were deleted individually or in combination by using CRISPR/Cas9-mediated genome editing to obtain PpKT-D4187, PpKT-D1780, and PpKT-D4187/1780 (Fig. S4). The pSEVA::*P*_*46*_-*pig* was then introduced into these mutant strains to generate PpKT16, PpKT17, and PpKT18, respectively. In comparison to PpKT05, a significant increase in prodigiosin production was observed in PpKT16, while no significant difference was observed in PpKT17. The titer of prodigiosin in PpKT18 was decreased compared to PpKT05 (Fig. 5D). To exclude the possibility of polar effects potentially caused by the *PP*_*4187* mutation, genetic complementation was conducted by introducing a functional copy of *PP*_*4187* driven by its native promoter. HPLC analysis showed that prodigiosin production in the complementation strain PpKT19 was lower than that in PpKT05 (Fig. S5A), most likely attributed to the dosage effect of multiple *PP*_*4187* copies on the pSEVA-gRic6T plasmid. Moreover, the plasmid pSEVA::*P*_*46*_-*pig*-*P*_*rha*_-*bauA*-*mcrC* was also introduced into PpKT-D4187 and PpKT-D1780 to generate PpKT20 and PpKT21, respectively. Unexpectedly, the yields of prodigiosin in both recombinant strains were decreased dramatically (Fig. S5B). These data demonstrate that random chromosomal integration of transposon Tn5 is an efficient strategy for screening target genes to improve prodigiosin production.

### 3.6 Optimization of fermentation conditions for prodigiosin production

**Fig. 6:**
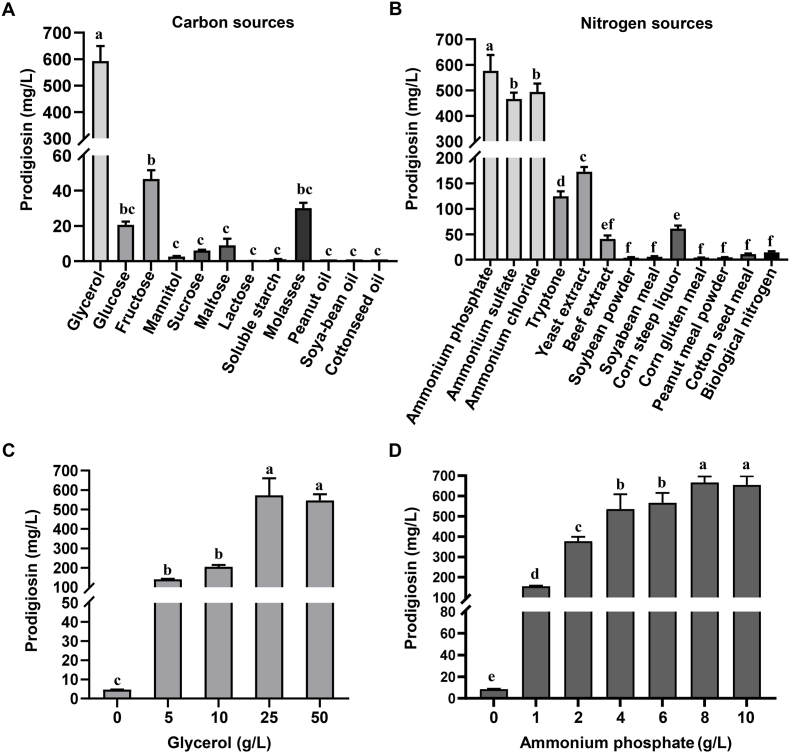
Prodigiosin production in PpKT16 cultivated in different fermentation media. (A) Yields of prodigiosin in PpKT16 cultivated with different carbon sources with 4 g/L ammonium phosphate. (B) Yields of prodigiosin in PpKT16 cultivated with different nitrogen sources with 25 g/L glycerol. (C) Effect of different glycerol concentrations on prodigiosin production with 4 g/L ammonium phosphate. (D) Effect of different ammonium phosphate concentrations on prodigiosin production with 25 g/L glycerol. Error bars represent standard deviations. Mean values with the same superscript letters are not significantly different, while those with different superscript letters are significantly different.

**Fig. 7:**
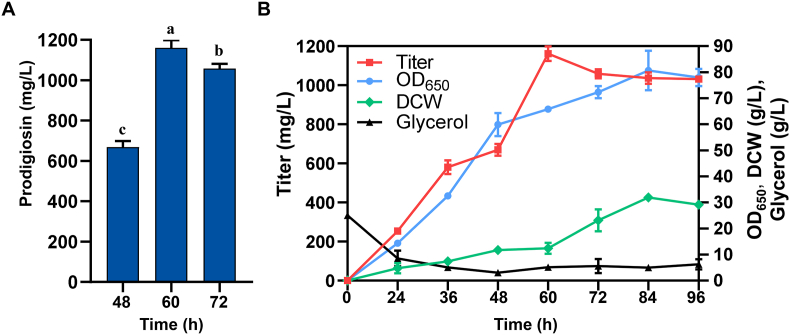
Fed-batch fermentation of PpKT16 in a 5 L stirred-tank bioreactor. (A) Prodigiosin production during fermentation in modified RK medium with glycerol (25 g/L) and ammonium phosphate (8 g/L) at three time-points. (B) Time profile of fed-batch cultivation with glycerol as the sole carbon source. The Y-axis shows the titers of prodigiosin, the X-axis represents different time points. Error bars represent standard deviations. Mean values with the same superscript letters are not significantly different, while those with different superscript letters are significantly different. Symbols: red square, prodigiosin titer (mg/L); blue circle, OD_650_; green diamond, dry cell weight (DCW, g/L); black triangle, glycerol residual concentration (g/L).

To achieve maximal level of prodigiosin production, it is of great importance to optimize the culture media. We selected PpKT16 to investigate the effects of different carbon and nitrogen sources on prodigiosin production, as this strain eliminates the need for rhamnose supplementation, a requirement associated with PpKT10. HPLC analysis showed that PpKT16 accumulated the highest level of prodigiosin when glycerol was used as the carbon source. In contrast, prodigiosin production was significantly reduced when the strain was cultured in fermentation media supplemented with alternative carbon sources, including various sugars such as glucose, lactose, and soluble starch, as well as several oils rich in unsaturated fatty acids such as peanut oil (Fig. 6A). Similarly, PpKT16 exhibited remarkable differences in prodigiosin yield when utilizing different nitrogen sources. Prodigiosin production was notably higher with inorganic nitrogen sources, including ammonium phosphate, ammonium sulfate, and ammonium chloride, with ammonium phosphate being the most effective. By contrast, prodigiosin production decreased substantially when organic nitrogen sources were used in the fermentation media, such as tryptone, yeast extract, beef extract, and soybean powder (Fig. 6B). Subsequently, concentrations of glycerol and ammonium phosphate were further optimized to enhance prodigiosin production. We noticed that a maximum increase was observed with glycerol at 25 g/L and ammonium phosphate at 8 g/L, reaching a yield of 665 mg/L (Fig. 6C and D). Furthermore, we also evaluated the effect of supplementing putative biosynthetic precursors (pyruvate, proline, and 2-octenal) on production. Unfortunately, no obvious increase was observed with supplementation of these substrates (Fig. S6). To further evaluate the productivity of the engineered strain PpKT16, a scale-up fermentation was conducted in a 5 L stirred-tank bioreactor. A maximum yield of 1161 mg/L was observed after cultivation at 60 h (Fig. 7A). Fed-batch fermentation was adopted to maintain glycerol availability under continuous aeration conditions. After 36 h of fermentation, the glycerol concentration decreased to 10 g, prompting the initiation of glycerol feeding to sustain a concentration of approximately 10 g from 36 h to 96 h. Cell growth entered the stationary phase after 72 h of cultivation. In contrast, the prodigiosin titer continued to increase until 60 h. These results indicate that maintaining glycerol at around 10 g was sufficient to support prodigiosin biosynthesis without stimulating excessive cell growth (Fig. 7B). Overall, the strategy effectively supported ongoing prodigiosin biosynthesis during this period.

## 4 Discussion

*S. marcescens* is frequently isolated from both natural environments and hospital settings, and it is best-known as the producer of prodigiosin. Prodigiosin has attracted increasing attention due to its diverse beneficial properties, including its role as a potent anticancer agent that induces apoptosis, an antibiotic against multi pathogens, and an immunosuppressive compound [49,50]. Over the past few decades, extensive research has focused on strain improvement of natural prodigiosin-producing bacteria to enhance prodigiosin yield. The primary strategies employed include random mutagenesis and genetic engineering approaches. The latter encompasses the modification of individual genes within the *pig* gene cluster to enhance their transcriptional stability, as well as the targeted engineering of regulatory factors involved in its biosynthesis [[51], [52], [53]]. Furthermore, the optimization of fermentation conditions has also significantly increased the prodigiosin production, including the addition of trace elements and precursor amino acids, and the use of low-cost substrates like cassava wastewater, peanut meal, and olive oil as alternative carbon and nitrogen sources [[53], [54], [55]]. These efforts have led to the development of high-yielding strains capable of producing prodigiosin at gram-scale levels. Despite these advances, the use of *S. marcescens* for industrial-scale prodigiosin production remains challenging. First, as a conditionally pathogenic bacterium, *S. marcescens* poses potential biosafety concerns during large-scale cultivation. Furthermore, its inherent capacity to synthesize a diverse array of secondary metabolites complicates the downstream separation and purification of prodigiosin. This not only increases the technical difficulty of isolating high-purity prodigiosin but also undermines the cost-effectiveness of its manufacturing, limiting its practical application in industrial settings.

To address these limitations, heterologous production strategies have emerged as promising alternatives. Previous studies demonstrated that *P. putida* KT2440 holds significant potential as a heterologous host for prodigiosin production [29,56]. In this study, we isolated three *S. marcescens* strains and focused on the heterologous expression of the *pig* gene cluster in *P. putida* KT2440. Moreover, multiple metabolic engineering strategies were employed to enhance prodigiosin yield. Notably, two strategies contributed to significant improvement in prodigiosin titers. The first involves promoter engineering, which relies on the screening of various well-characterized promoters. To our knowledge, a key limitation of this strategy lies in the availability of a diverse pool of promoters. The second strategy entails constructing a Tn5-tagged mutant library. Subsequent screening of this library enables the identification of specific negative regulatory target genes, whose deletion unlocks the biosynthetic potential of the surrogate host. A key limitation of this approach, however, is the inability to identify positive regulatory target genes. We found that promoter engineering of the *pig* gene cluster, combined with knockout of the negative effector in *P. putida* KT2440, enhanced prodigiosin production. When cultivated in a 5 L fermenter, the engineered strain exhibited the maximum prodigiosin yield at 1161 mg/L.

Malonyl-CoA is a key precursor for the biosynthesis of PKS derived metabolites, as it serves as the building block for the polyketide moiety of prodigiosin [13,57]. In a previous study, a synthetic pathway for malonyl-CoA formation was designed and introduced into multiple model and non-model microorganisms—including *E. coli*, *S. gilvosporeus* and *Saccharopolyspora spinosa*—and this modification significantly enhanced the production of various value-added malonyl-CoA-derived products (MDPs), such as those belonging to phenol, quinone, alkene, aminoglycoside and macrolide polyketide families [33]. In this study, this pathway was introduced into *P. putida* KT2440 to boost prodigiosin production. Three different promoters were selected to drive the expression of the artificial pathway in this strain: the constitutive promoter *P*_*46*_, the cell density‐dependent inducible promoter *Rox3061* and the rhamnose-inducible promoter *rha*. This approach yielded three engineered strains: PpKT08, PpKT09, and PpKT10. A significant reduction in prodigiosin production was observed in strains PpKT09 and PpKT10 relative to PpKT08. However, a marked increase in prodigiosin production was detected in PpKT10 when the strain was cultured in RK medium supplemented with 0.001 % rhamnose (Fig. 4C). These findings indicate that precise modulation of the malonyl-CoA pathway is critical for enhancing prodigiosin yields. It is noteworthy that malonyl-CoA acts as a central metabolite and serves as the universal precursor for fatty acid and polyketide biosynthesis. Given the complexity associated with regulating intracellular malonyl-CoA levels, further efforts should focus on redirecting metabolic flux of malonyl-CoA toward prodigiosin biosynthesis.

Previous studies have also shown that the addition of proline, glutamic acid, alanine, and serine in the medium can promote prodigiosin production [58,59]. 2‐octenal is another key precursor of prodigiosin, primarily derived from fatty acid oxidation [60]. Unfortunately, our attempt to supplement the fermentation medium with proline and 2-octenal failed to enhance prodigiosin production. The metabolic context of *P. putida* KT2440 likely differs from that of prodigiosin's native producers; alternatively, the uptake or conversion of these supplemented precursors may have been inefficient under the current fermentation conditions. Future studies could explore co-engineering the pathways for these precursors, such as overexpressing proline synthetase or fatty acid synthase, to establish a more balanced supply of all building blocks, which may in turn drive further improvements in prodigiosin yields.

Random mutagenesis is an effective technique for identifying targets that negatively affect prodigiosin biosynthesis, and such mutagenesis approaches have been successfully applied in *S. marcescens* [52,61]. We herein identified two mutants in *P. putida* KT2440 that exhibit enhanced prodigiosin production, with these phenotypes associated with mutations in genes encoding dihydrolipoyl dehydrogenase and mannosyltransferase, respectively. In *E. coli*, dihydrolipoyl dehydrogenase is a component of the pyruvate dehydrogenase complex (PDHc), which catalyzes the conversion of pyruvate to acetyl-CoA [62]. We speculate that disruption of dihydrolipoyl dehydrogenase may lead to the accumulation of pyruvate, an important precursor for prodigiosin synthesis. While the precise role of mannosyltransferase in prodigiosin biosynthesis remains unclear, this enzyme is known to be implicated in outer membrane biogenesis [48]. Thus, we hypothesize that deletion of the corresponding gene may alter cell morphology in *P. putida* KT2440. Notably, a previous study demonstrated that the formation of OMVs in *P. putida* KT2440 correlates with increased production of various secondary metabolites, including prodigiosin [28]. Our findings indicate that targeted mutagenesis of central metabolic and membrane-associated genes can significantly enhance prodigiosin yields. However, additional genome-wide investigations are required to identify genes that positively regulate prodigiosin biosynthesis.

## 5 Conclusion

In this study, prodigiosin over-producing strains were constructed using a stepwise strategy including heterologous expression, gene cluster engineering, and genetic manipulation of the surrogate host. Specifically, two key modifications contributed to significant increases in prodigiosin titers: engineering of the *pig* gene cluster with constitutive promoters, and deletion of a gene that exerts a negative effect on prodigiosin biosynthesis in *P. putida* KT2440. The introduction of an engineered gene cluster under the control of the *P*_*46*_ promoter into *PP_4187* deletion mutant resulted in the generation of PpKT16. Prodigiosin production in PpKT16 was detected at 587 mg/L, which was slightly lower than the 601 mg/L observed in PpKT10 upon rhamnose induction. However, PpKT16 is more suitable for further industrial scale-up, as it eliminates the requirement for exogenous rhamnose supplementation. Subsequent optimization of fermentation conditions with PpKT16 further boosted prodigiosin production to 665 mg/L. Notably, the maximum titer of prodigiosin reached 1161 mg/L when the engineered strain PpKT16 was cultivated in a 5 L stirred-tank bioreactor. These results clearly demonstrate that combined genetic and metabolic engineering approaches can substantially boost prodigiosin biosynthesis in a heterologous host. However, further studies are required to fully elucidate how the identified gene deletions modulate metabolic flux toward prodigiosin biosynthesis. Furthermore, extending these engineering strategies to other secondary metabolite pathways will be critical for enabling broader application in the large-scale production of high-value natural products.

## CRediT authorship contribution statement

**Yuxin Zhang:** Writing – review & editing, Validation, Methodology, Investigation. **Meiyan Wang:** Validation, Methodology, Investigation. **Kaijie Dou:** Validation, Investigation. **Ruizhi Zhang:** Validation, Methodology. **Chunfang Wang:** Methodology, Investigation. **Xiaoying Bian:** Methodology, Conceptualization. **Jun Si:** Writing – review & editing, Supervision, Conceptualization. **Guoqing Niu:** Writing – review & editing, Writing – original draft, Supervision, Funding acquisition.

## Funding

This study was supported in part by a grant from the 10.13039/501100012166National Key Research and Development Program of China (No. 2023YFD1700700), and a grant from the 10.13039/501100001809National Natural Science Foundation of China (No. 32370077).

## Declaration of competing interest

The authors declare that they have no known competing financial interests or personal relationships that could have appeared to influence the work reported in this paper.
