# Supplementary material for: High-level prodigiosin production in Pseudomonas putida enabled by combinatorial metabolic engineering
Source: Synth Syst Biotechnol. 2026 Jan 30;13:98–108. doi: 10.1016/j.synbio.2026.01.015 (PMC12874325; doi:10.1016/j.synbio.2026.01.015)
Supplement: Multimedia component 1 [file mmc1.docx]

Supplementary Material

**High-Level Prodigiosin Production in *Pseudomonas putida* Enabled by Combinatorial Metabolic Engineering**

Yuxin Zhang^1, 2^, Meiyan Wang^1^, Kaijie Dou^1^, Ruizhi Zhang^3^, Chunfang Wang^4^, Xiaoying Bian^5^, Jun Si^6,*^ and Guoqing Niu^1, 2,*^

^1^ College of Agronomy and Biotechnology, Southwest University, Chongqing, 400715, China

^2^ Institute of Biotechnology, Shanxi University, Taiyuan, 030006, Shanxi, China

^3^ College of Pharmaceutical Sciences and Chinese Medicine, Southwest University, Chongqing, 400715, China

^4^ Southwest University Hospital, Chongqing 400715, China

^5^ Helmholtz International Lab for Anti-infectives, Shandong University-Helmholtz Institute of Biotechnology, State Key Laboratory of Microbial Technology, Shandong University, Qingdao, Shandong 266237, China.

^6^ College of Horticulture and Landscape Architecture, Southwest University, Chongqing 400715, China.

***Corresponding authors**

Guoqing Niu

E-mail address: niu062376@swu.edu.cn

Jun Si

E-mail address: sijun@swu.edu.cn

# Supplementary Figures


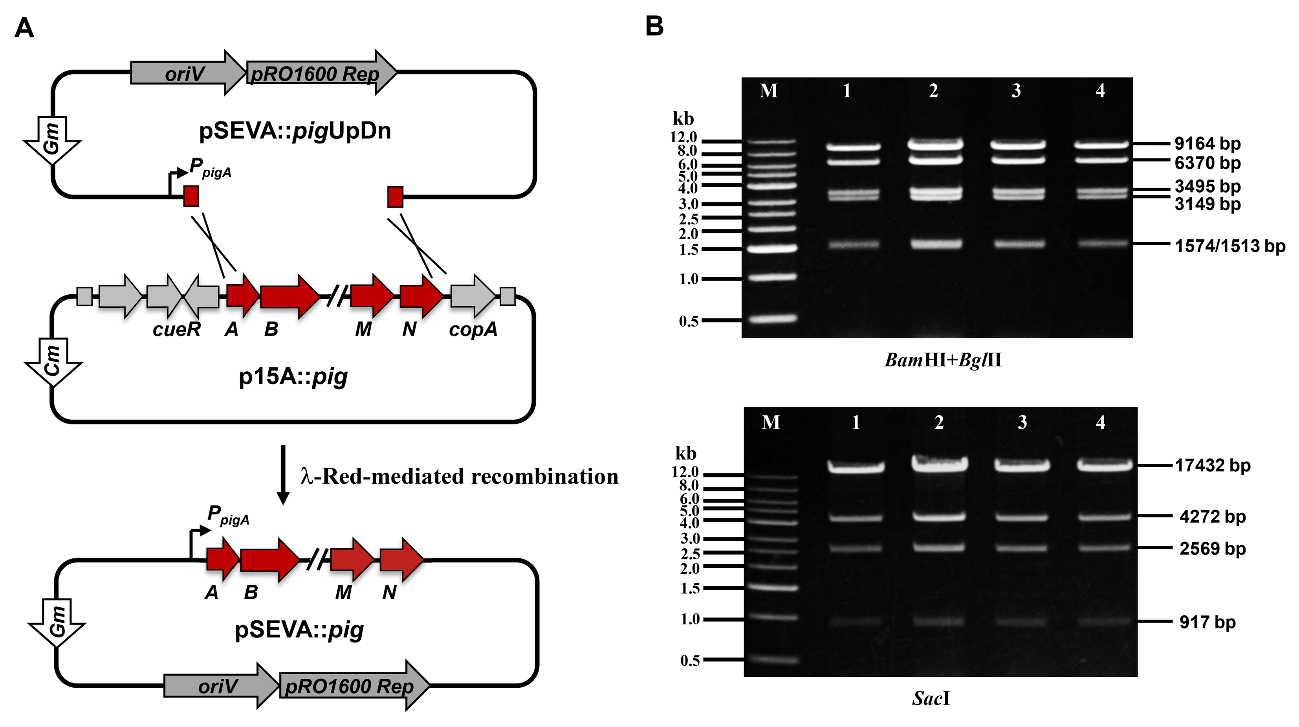


**Figure S1. A simplified schematic diagram for the construction of** **pSEVA::*pig*.** (A) The procedure for constructing pSEVA::*pig* was described in detail in the Materials and Methods. The *pig* gene cluster was transferred into pSEVA *via* λ-Red-mediated recombination with a *Xho*I linearized plasmid to obtain pSEVA::*pig*. (B) Gel electrophoresis results of restriction digestion either with *Bam*HI and *Bgl*II (the upper gel) or with *Sac*I (the lower gel). M represents the DNA marker, and 1-4 represent different samples of the engineered gene clusters with indicated fragment sizes in base pairs.


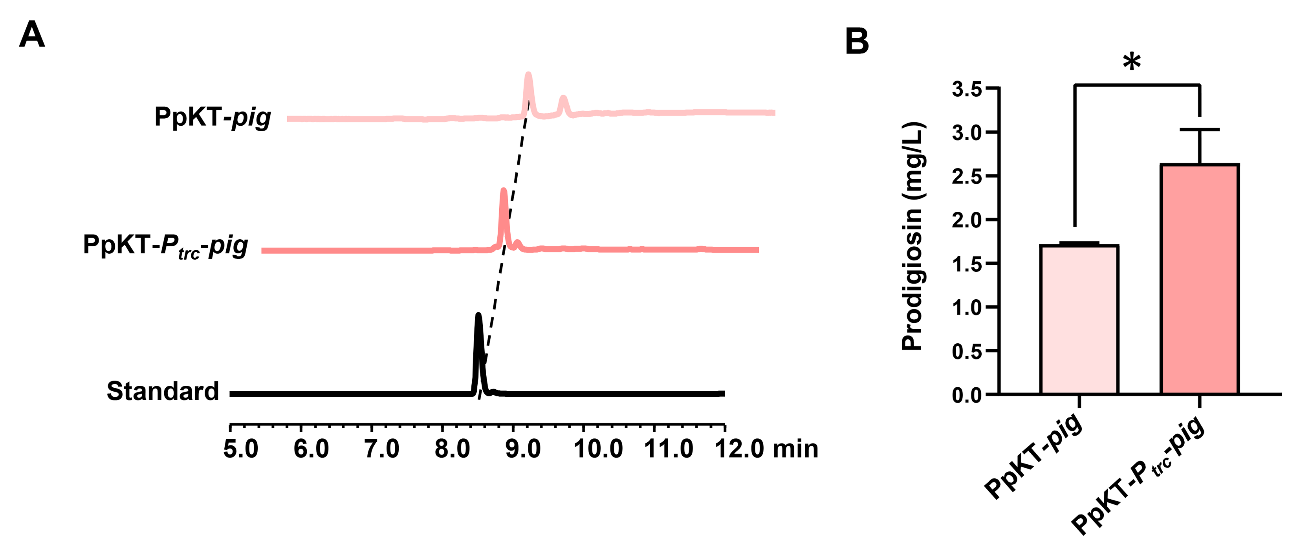


**Figure S2. Assessment of prodigiosin production in PpKT-*pig* and PpKT-*P_trc_*-*pig*.** (A) HPLC analysis of extracts from PpKT-*pig* and PpKT-*P_trc_*-*pig* compared to the prodigiosin standard. (B) Prodigiosin yield in fermentation broths from PpKT-*pig* and PpKT-*P_trc_*-*pig*. PpKT-*pig* was cultivated in RK medium, while PpKT-*P_trc_*-*pig* was cultivated in RK medium supplemented with 0.05 mM IPTG. Error bars represent standard deviations. “*” represents significant difference according to statistical analysis (*P* < 0.05).


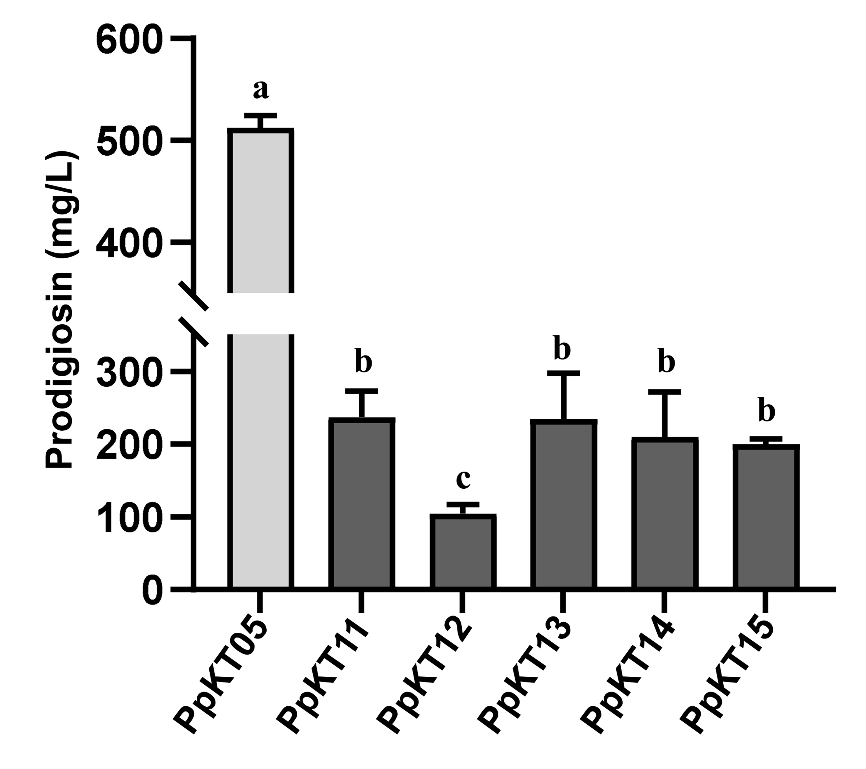


**Figure S3. Production of** **prodigiosin in PpKT05 and** **PpKT11-15.** Comparison of prodigiosin levels in PpKT05 and PpKT11-15. PpKT05: *P. putida* KT2440 harboring the pSEVA::*P_46_*-*pig*; PpKT11-PpKT15: *P. putida* KT2440*-*derived deletion mutants (*PP_5245*, *PP*_*2148*, *PP*_*2080*, *PP*_*2126*, and *PP*_*1182*) with each strain harboring pSEVA::*P_46_*-*pig*. Error bars represent standard deviations. Mean values with the same superscript letters are not significantly different, while those with different superscript letters are significantly different.


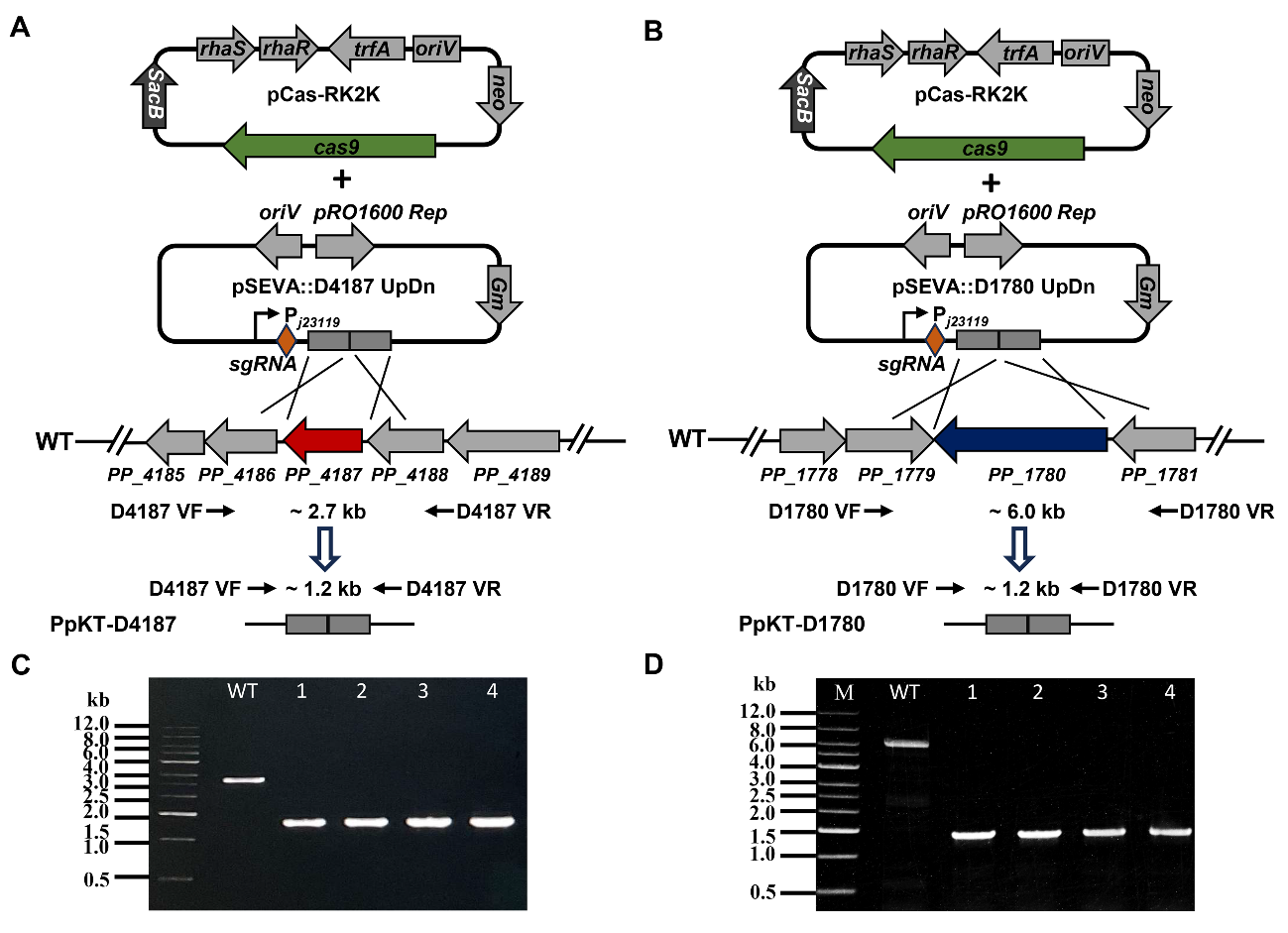


**Figure S4. Schematic illustrations of CRISPR/Cas9-mediated genome engineering for the construction of** **PpKT-D4187 and PpKT-D1780.** (A) Schematic representation of CRISPR/Cas9-mediated knock-out of *PP*_*4187* to generate PpKT-D4187. (B) Schematic representation of CRISPR/Cas9-mediated knock-out of *PP*_*1780* to generate PpKT-D1780. (C) Verification of PpKT-D4187 by PCR amplifications. (D) Verification of PpKT-D1780 by PCR amplifications. The expected sizes of PCR amplicons are as indicated. “kb” represents kilobase, and “M” denotes DNA Ladder. Four transformants (1-4) of each engineered strain were randomly selected for DNA extractions and subsequent PCR amplifications. The parental strain (WT) used in CRISPR/Cas9-mediated engineering was included to serve as a control.


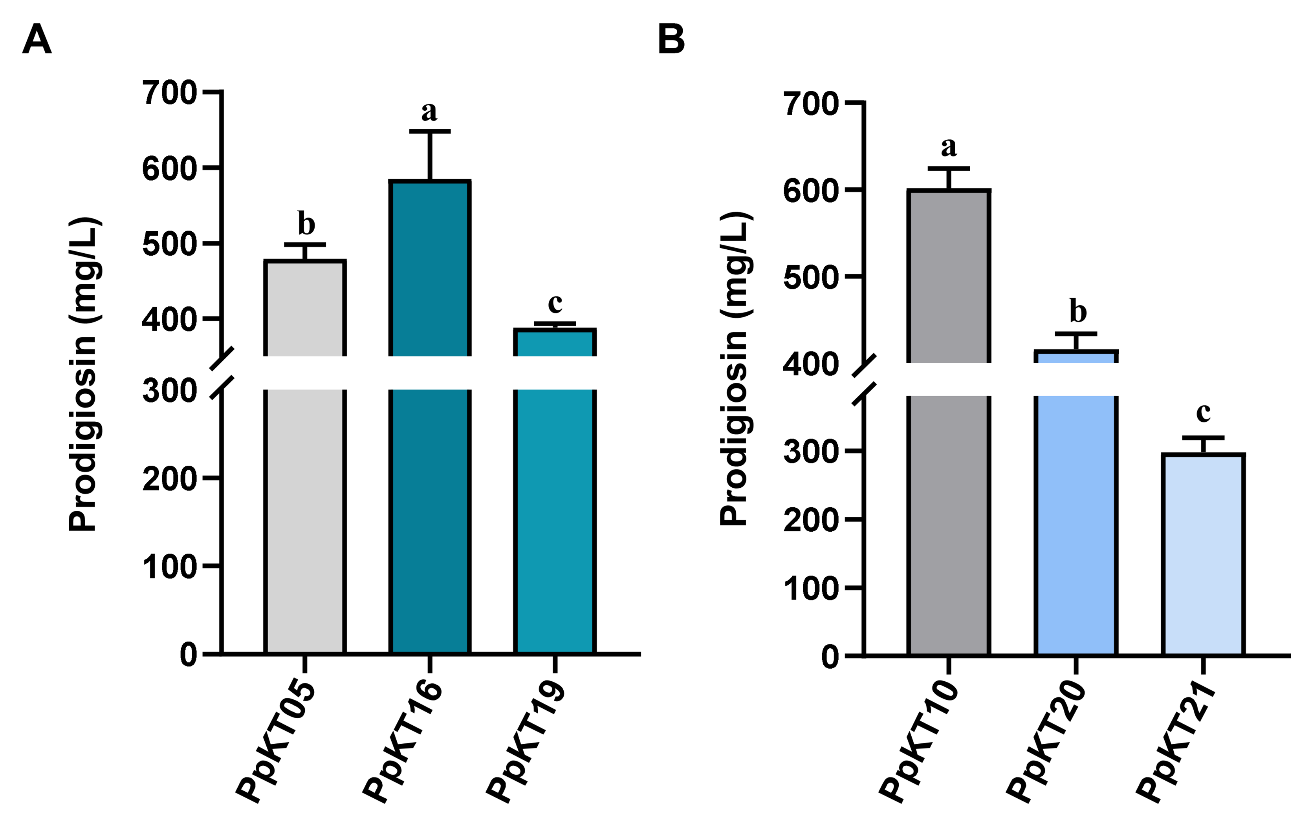


**Figure S5. Production of prodigiosin in PpKT05 and** **its derivatives.** (A) Comparison of prodigiosin levels in PpKT05, PpKT16, and PpKT19. PpKT05: *P. putida* KT2440 harboring the pSEVA::*P_46_*-*pig*; PpKT16: PpKT-D4187 (*PP*_*4187* deletion mutant) harboring the pSEVA::*P_46_*-*pig*; PpKT19: the complementation strain of PpKT-D4187 harboring the pSEVA::*P_46_*-*pig*-C4187. The three strains were cultivated in RK medium. (B) Comparison of prodigiosin levels in PpKT10, PpKT20, and PpKT21. PpKT10: *P. putida* KT2440 harboring the pSEVA::*P_46_*-*pig*-*P_rha_*-*bauA*-*mcrC*; PpKT20: PpKT-D4187 harboring pSEVA::*P_46_*-*pig*-*P_rha_*-*bauA*-*mcrC*; PpKT21: PpKT-D1780 harboring pSEVA::*P_46_*-*pig*-*P_rha_*-*bauA*-*mcrC*. The three strains were cultivated in RK medium supplemented with 0.001% rhamnose. Error bars represent standard deviations. Mean values with the same superscript letters are not significantly different, while those with different superscript letters are significantly different.


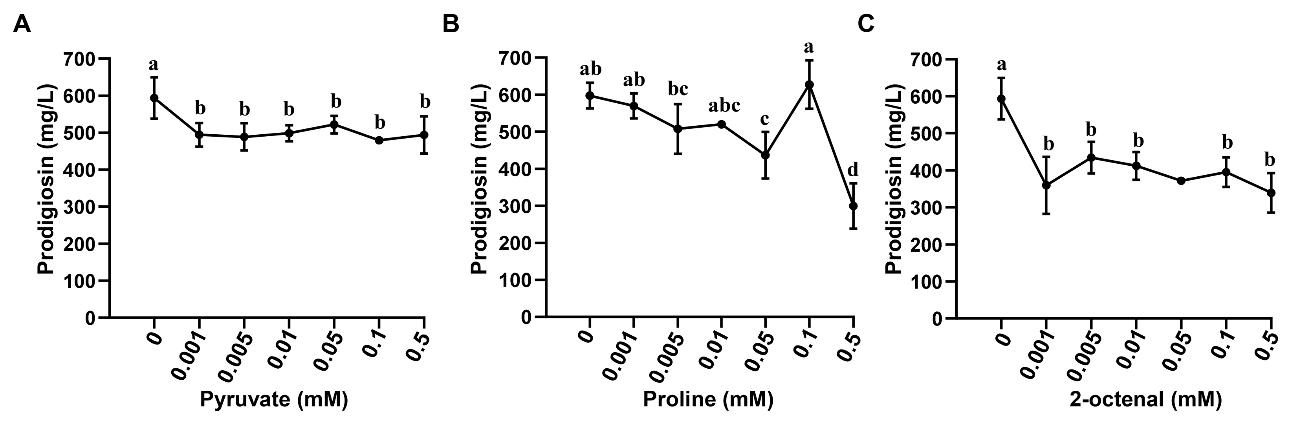


**Figure S6. Prodigiosin production by PpKT16 in fermentation supplemented with different precursors.** Prodigiosin yield in RK media supplemented with different concentrations of pyruvate (A), proline (B), and 2-octenal (C). Error bars represent standard deviations. Mean values with the same superscript letters are not significantly different, while those with different superscript letters are significantly different.

# Supplementary Tables

**Table S1.** Bacterial strains used in this study.

| **Strains** | **Relevant Characteristics** | **Reference/Source** |
| --- | --- | --- |
| ***Serratia* Strains** |  |  |
| *S. marcescens* BoR121 | Wild-type, prodigiosin producer | [1] |
| *S. marcescens* BoR4-1 | Wild-type, prodigiosin producer | This work |
| *S. marcescens* BoR4-11 | Wild-type, prodigiosin producer | This work |
| ***E. coli* Strains** |  |  |
| DH5α | F^–^, φ80*lac*ZΔM15, Δ(*lac*ZYA-*arg*F) U169, *rec*A1, *end*A1, *hsd*R17 (r_K_^–^, m_K_^+^), *pho*A, *sup*E44, λ^–^, *thi*-1, *gyr*A96, *rel*A1 | Thermo Fisher Scientific |
| BW25113 | K-12 derivative; Δ*araBAD* Δ*rhaBAD* | [2] |
| S17-1 λpir | *res*^–^, *pro*A, *mod*^+^, *thi*-1, integrated copy of RP4 | Beyotime Biotech Inc |
| ***Pseudomonas* Strains** |  |  |
| *P. putida* KT2440 | Wild-type, mt-2 derivative cured of the TOL plasmid pWW0 | [3] |
| PpKT-*pig* | *P. putida* KT2440 containing  pSEVA::*pig*, Gen^R^ | This work |
| PpKT-*P_trc_*-*pig* | *P. putida* KT2440 containing  pSEVA::*P_trc_*-*pig*, Gen^R^ | This work |
| PpKT01 | *P. putida* KT2440 containing  pSEVA::*P_cgtrc_*-*pig*, Gen^R^ | This work |
| PpKT02 | *P. putida* KT2440 containing  pSEVA::BG42-*pig*, Gen^R^ | This work |
| PpKT03 | *P. putida* KT2440 containing  pSEVA::*P_rpsJ12_*-*pig*, Gen^R^ | This work |
| PpKT04 | *P. putida* KT2440 containing  pSEVA::*P_35_*-*pig*, Gen^R^ | This work |
| PpKT05 | *P. putida* KT2440 containing  pSEVA::*P_46_*-*pig*, Gen^R^ | This work |
| PpKT06 | *P. putida* KT2440 containing  pSEVA::LvaR/*P_lvaA_*-*pig*, Gen^R^ | This work |
| PpKT07 | *P. putida* KT2440 containing  pSEVA::*P_Rox3061_*-*pig*, Gen^R^ | This work |
| PpKT08 | *P. putida* KT2440 containing  pSEVA::*P_46_*-*pig*-*P_46_*-*bauA*-*mcrC*, Gen^R^ | This work |
| PpKT09 | *P. putida* KT2440 containing  pSEVA::*P_46_*-*pig*-*P_Rox3061_*-*bauA*-*mcrC*, Gen^R^ | This work |
| PpKT10 | *P. putida* KT2440 containing  pSEVA::*P_46_*-*pig*-*P_rha_*-*bauA*-*mcrC*, Gen^R^ | This work |
| PpKT-D5245 | A derivative of *P. putida* KT2440 deficient in *PP*_*5245* | This work |
| PpKT-D2148 | A derivative of *P. putida* KT2440 deficient in *PP*_*2148* | This work |
| PpKT-D2080 | A derivative of *P. putida* KT2440 deficient in *PP*_*2080* | This work |
| PpKT-D2126 | A derivative of *P. putida* KT2440 deficient in *PP*_*2126* | This work |
| PpKT-D1182 | A derivative of *P. putida* KT2440 deficient in *PP*_*1182* | This work |
| PpKT-D4187 | A derivative of *P. putida* KT2440 deficient in *PP*_*4187* | This work |
| PpKT-D1780 | A derivative of *P. putida* KT2440 deficient in *PP*_*1780* | This work |
| PpKT-D4187/1780 | A derivative of *P. putida* KT2440 deficient in *PP*_*4187* and *PP_1780* | This work |
| PpKT11 | PpKT-D5245 containing pSEVA::*P_46_*-*pig*, Gen^R^ | This work |
| PpKT12 | PpKT-D2148 containing pSEVA::*P_46_*-*pig*, Gen^R^ | This work |
| PpKT13 | PpKT-D2080 containing pSEVA::*P_46_*-*pig*, Gen^R^ | This work |
| PpKT14 | PpKT-D2126 containing pSEVA::*P_46_*-*pig*, Gen^R^ | This work |
| PpKT15 | PpKT-D1182 containing pSEVA::*P_46_*-*pig*, Gen^R^ | This work |
| PpKT16 | PpKT-D4187 containing pSEVA::*P_46_*-*pig*, Gen^R^ | This work |
| PpKT17 | PpKT-D1780 containing pSEVA::*P_46_*-*pig*, Gen^R^ | This work |
| PpKT18 | PpKT-D4187/1780 containing pSEVA::*P_46_*-*pig*, Gen^R^ | This work |
| PpKT19 | PpKT-D4187 containing pSEVA::*P_46_*-*pig*-C4187, Gen^R^ | This work |
| PpKT20 | PpKT-D4187 containing pSEVA::*P_46_*-*pig*-*P_rha_*-*bauA*-*mcrC*, Gen^R^ | This work |
| PpKT21 | PpKT-D1780 containing pSEVA::*P_46_*-*pig*-*P_rha_*-*bauA*-*mcrC*, Gen^R^ | This work |

Gen^R^: gentamicin resistance.

**Table S2.** Plasmids used in this study.

| **Plasmids** | **Relevant Characteristics** | **Reference/Source** |
| --- | --- | --- |
| pIJ790 | λ-RED (*gam, bet, exo*), *cat*, *araC*, *rep101*^ts^ | [4] |
| pSEVA-gRic6T | Derived from pSEVA-gRNA, pJ23119-sgRNA-nicC (*PP*_*3944*) | [5] |
| pCAS-RK2K | *oriRK2*, Kan^R^, *Pcas*-Cas9, *P_araB_*-Red, *P_rhaB_*-sgRNA-pRO1600, SacB | [5] |
| pCDF-*bauA*-*mcrC* | pCDF-duet carrying *bauA* and *mcrC* driven by T7, Sm^R^ | [6] |
| pSEVA::*pig*UpDn | A derivative of pSEVA-gRic6T containing upstream and downstream regions of the *pig* gene cluster, Gen^R^ | This work |
| pSEVA::*pig* | A derivative of pSEVA-gRic6T containing the entire *pig* gene cluster, Gen^R^ | This work |
| pSEVA::*P_trc_*-*pig* | A derivative of pSEVA-gRic6T containing the *pig* gene cluster driven by *P_trc_*, Gen^R^ | This work |
| pSEVA::*P_cgtrc_*-*pig* | A derivative of pSEVA-gRic6T containing the *pig* gene cluster driven by *P_cgtrc_*, Gen^R^ | This work |
| pSEVA::BG42-*pig* | A derivative of pSEVA-gRic6T containing the *pig* gene cluster driven by BG42, Gen^R^ | This work |
| pSEVA::*P_rpsJ12_*-*pig* | A derivative of pSEVA-gRic6T containing the *pig* gene cluster driven by *P_rpsJ12_*, Gen^R^ | This work |
| pSEVA::*P_35_*-*pig* | A derivative of pSEVA-gRic6T containing the *pig* gene cluster driven by *P_35_*, Gen^R^ | This work |
| pSEVA::*P_46_*-*pig* | A derivative of pSEVA-gRic6T containing the *pig* gene cluster driven by *P_46_*, Gen^R^ | This work |
| pSEVA::LvaR/*P_lvaA_*-*pig* | A derivative of pSEVA-gRic6T containing the *pig* gene cluster driven by LvaR/*P_lvaA_*, Gen^R^ | This work |
| pSEVA::*P_Rox3061_*-*pig* | A derivative of pSEVA-gRic6T containing the *pig* gene cluster driven by *P_Rox3061_*, Gen^R^ | This work |
| pSEVA::*P_46_*-*pig*-*P_46_*-*bauA*-*mcrC* | A derivative of pSEVA::*P_46_*-*pig* containing *bauA* and *mcrC* driven by *P_46_*, Gen^R^ | This work |
| pSEVA::*P_46_*-*pig*-*P_Rox3061_*-*bauA*-*mcrC* | A derivative of pSEVA::*P_46_*-*pig* containing *bauA* and *mcrC* driven by *P_Rox3061_*, Gen^R^ | This work |
| pSEVA::*P46*-*pig*-*P_rha_*-*bauA*-*mcrC* | A derivative of pSEVA::*P_46_*-*pig* containing *bauA* and *mcrC* driven by *P_rha_*, Gen^R^ | This work |
| pSEVA::D5245 UpDn | A derivative of pSEVA-gRic6T containing sgRNA targeting *PP*_*5245* and upstream and downstream regions of *PP*_*5245*, Gen^R^ | This work |
| pSEVA::D2148 UpDn | A derivative of pSEVA-gRic6T containing sgRNA targeting *PP*_*2148* and upstream and downstream regions of *PP*_*2148*, Gen^R^ | This work |
| pSEVA::D2080 UpDn | A derivative of pSEVA-gRic6T containing sgRNA targeting *PP*_*2080* and upstream and downstream regions of *PP*_*2080*, Gen^R^ | This work |
| pSEVA::D2126 UpDn | A derivative of pSEVA-gRic6T containing sgRNA targeting *PP*_*2126* and upstream and downstream regions of *PP*_*2126*, Gen^R^ | This work |
| pSEVA::D1182 UpDn | A derivative of pSEVA-gRic6T, containing sgRNA targeting *PP*_*1182* and upstream and downstream regions of *PP*_*1182*, Gen^R^ | This work |
| pSEVA::D1780 UpDn | A derivative of pSEVA-gRic6T containing sgRNA targeting *PP*_*1780* and upstream and downstream regions of *PP*_*1780*, Gen^R^ | This work |
| pSEVA::D4187 UpDn | A derivative of pSEVA-gRic6T containing sgRNA targeting *PP*_*4187* and upstream and downstream regions of *PP*_*4187*, Gen^R^ | This work |
| pSEVA::*P_46_*-*pig*-C4187 | A derivative of pSEVA::*P_46_*-*pig* containing the coding region of *PP_4187* and its upstream promoter, Gen^R^ | This work |

Gen^R^: gentamicin resistance; Kan^R^: kanamycin resistance; Sm^R^: streptomycin resistance.

**Table S3.** Primers used in this study.

| **Primers** | **Sequence (5′-3′)** ^a, b^ | **Purpose** |
| --- | --- | --- |
| pig UpF | aattGAATTCTTTTTCCTCCGGAATGCTCCTG | Construction of pSEVA::*pig* |
| pig UpR | aattCTCGAGATGTCGGCAATCGGCGCGTT | Construction of pSEVA::*pig* |
| pig DnF | aattCTCGAGCCCCAACGCGGCAGCGCGGTT | Construction of pSEVA::*pig* |
| pig DnR | aattAAGCTTCGGCATCGCCGTACACTTTC | Construction of pSEVA::*pig* |
| trc pF | *TCGTTTTATTTGATGCCTTTAA*TTAATCACTGCCCGCTTTCCAGTC | Construction of pSEVA::*P_trc_*-*pig* |
| trc pR | *TGATAAGTTAAAATCCAT*ATGTATATCTCCTTCTTAAAAG | Construction of pSEVA::*P_trc_*-*pig* |
| cgtrc pF | aattGAATTCTTGACAATTAATCATCCGGC | Construction of pSEVA::*P_cgtrc_*-*pig* |
| cgtrc pR | GGTCTGTTTCCTGTGTGAAA | Construction of pSEVA::*P_cgtrc_*-*pig* |
| BG42 pF | aattGAATTCGCCCATTGACAAGGCTCTCGCGGCCAGGTATAATTGCACGA*ATGGATTTTAACTTATCAAG* | Construction of pSEVA::BG42-*pig* |
| rpsJ12 pF | aattGAATTCTCACTCGACCGATTGAAAAA | Construction of pSEVA::*P_rpsJ12_*-*pig* |
| rpsJ12 pR | TGGATTTCAGACTCCAGGCTG | Construction of pSEVA::*P_rpsJ12_*-*pig* |
| 35 pF | aattGAATTCGCCGTGACAGACAGCCCGTG | Construction of pSEVA::*P_35_*-*pig* |
| 35 pR | *TGATAAGTTAAAATCCAT*GTCATCGACTCCTGGCGCAA | Construction of pSEVA::*P_35_*-*pig* |
| 46 pF | aattGAATTCAGGCCCTTCTCCAGTTTTTCTTC | Construction of pSEVA::*P_46_*-*pig* |
| 46 pR | *TGATAAGTTAAAATCCAT*GCCGTTTTCCTCGCAGGCTG | Construction of pSEVA::*P_46_*-*pig* |
| LvaR pF | aattGAATTCTCAATTGGCAGATCGCAAGC | Construction of pSEVA::LvaR/*P_lvaA_*-*pig* |
| LvaR pR | *TGATAAGTTAAAATCCAT*GGTTCTGTAGGCCCTGCCTT | Construction of pSEVA::LvaR/*P_lvaA_*-*pig* |
| Rox3061 pF | aattGAATTCGCCTCCTTTCGTGTTTCGC | Construction of pSEVA::*P_Rox3061_*-*pig* |
| Rox3061 pR | *TGATAAGTTAAAATCCAT*GACCGACCTCTCAGGTTTTT | Construction of pSEVA::*P_Rox3061_*-*pig* |
| bauA F | *CTGAATTCATGGTGTCAA*TTAATTAACGGGATCTCGACGCTCTCCC | Construction of pSEVA::*P_46_*-*pig*-*P_46_*-*bauA*-*mcrC* |
| mcrC R | *TCGTTTTATTTGATGCCT*TTACACGGTAATCGCCCGTCC | Construction of pSEVA::*P_46_*-*pig*-*P_46_*-*bauA*-*mcrC* |
| 46 pF1 | *CTGAATTCATGGTGTCAA*TTAATTAAAGGCCCTTCTCCAGTTTTTCTTC | Construction of pSEVA::*P_46_*-*pig*-*P_46_*-*bauA*-*mcrC* |
| 46 pR1 | *GTTCAGCGGCTGATTCAT*GCCGTTTTCCTCGCAGGCTG | Construction of pSEVA::*P_46_*-*pig*-*P_46_*-*bauA*-*mcrC* |
| 46 pF2 | aattGAGCTCAGGCCCTTCTCCAGTTTTTCTTC | Construction of pSEVA::*P_46_*-*pig*-*P_46_*-*bauA*-*mcrC* |
| 46 pR2 | aattCATATGGCCGTTTTCCTCGCAGGCTG | Construction of pSEVA::*P_46_*-*pig*-*P_46_*-*bauA*-*mcrC* |
| Rox3061 pF1 | *CTGAATTCATGGTGTCAA*TTAATTAAGCCTCCTTTCGTGTTTCGCA | Construction of pSEVA::*P_46_*-*pig*-*P_Rox3061_*-*bauA*-*mcrC* |
| Rox3061 pR1 | *GTTCAGCGGCTGATTCAT*GACCGACCTCTCAGGTTTTT | Construction of pSEVA::*P_46_*-*pig*-*P_Rox3061_*-*bauA*-*mcrC* |
| Rox3061 pF2 | aattGAGCTCGCCTCCTTTCGTGTTTCGCA | Construction of pSEVA::*P_46_*-*pig*-*P_Rox3061_*-*bauA*-*mcrC* |
| Rox3061 pR2 | aattCATATGGACCGACCTCTCAGGTTTTT | Construction of pSEVA::*P_46_*-*pig*-*P_Rox3061_*-*bauA*-*mcrC* |
| rha pF1 | *CTGAATTCATGGTGTC*AATTAATCTTTCTGCGAATTGAG | Construction of pSEVA::*P_46_*-*pig*-*P_rha_*-*bauA*-*mcrC* |
| rha pR1 | *GTTCAGCGGCTGATT*CATATGTATATCTCCTTCTTAAAAGATCTTTTGAATTCTACGACCAGTCTAAAGAG | Construction of pSEVA::*P_46_*-*pig*-*P_rha_*-*bauA*-*mcrC* |
| rha pF2 | *GCGTAAGAATTCGAGCTC*ACTGGCCTCCTGATGTCGTC | Construction of pSEVA::*P_46_*-*pig*-*P_rha_*-*bauA*-*mcrC* |
| rha pR2 | *GGCGCTGAGATCTGCCAT*ATGTATATCTCCTTCTTAAAAG | Construction of pSEVA::*P_46_*-*pig*-*P_rha_*-*bauA*-*mcrC* |
| C4187 F | *CAGTCACGACGCGGCCGC*CCCTCTGGCTGCGTACCGTG | Construction of pSEVA::*P_46_*-*pig*-C4187 |
| C4187 R | *AAGTGTACGGCGATGCCGAAGCTT*TTAACGCTTCTTACGGTTGG | Construction of pSEVA::*P_46_*-*pig*-C4187 |
| D5245 sgRNA F1 | CCTAGGTATAATGCTAGCGCGATAGCCATTCAGTAACCGTTTTAGAGCTAGAAATAG | Construction of pSEVA::D5245 UpDn |
| D5245 sgRNA F2 | AATTGAATTCTTGACAGCTAGCTCAGT*CCTAGGTATAATGCTAGC* | Construction of pSEVA::D5245 UpDn |
| sgRNA R | aattGAGCTCCTCAAAAAAAGCACCGACTC | Construction of pSEVA::D5245 UpDn |
| D5245 Up F | aattGGATCCCTGAAAGCACGGGTGAAATG | Construction of pSEVA::D5245 UpDn |
| D5245 Up R | aattTCTAGAACCTCTTTCGCCTTGAATGC | Construction of pSEVA::D5245 UpDn |
| D5245 Dn F | aattTCTAGACGCTGCCTTCTTCGCGGGC | Construction of pSEVA::D5245 UpDn |
| D5245 Dn R | aattAAGCTTCAGTGCCGACACCAAACACC | Construction of pSEVA::D5245 UpDn |
| D2148 sgRNA F | aattCTTAAGCTGGCCGAACAGTGGGCTTTGTTTTAGAGCTAGAAATAGC | Construction of pSEVA::D2148 UpDn |
| D2148 sgRNA R | aattGGATCCCCGGGTACCGAGCTCCTCAA | Construction of pSEVA::D2148 UpDn |
| D2148 Up F | aattGGATCCCTCTTCACGGACGAACTGCT | Construction of pSEVA::D2148 UpDn |
| D2148 Up R | aattTCTAGAGGGAGATTGCACTCCAAGCG | Construction of pSEVA::D2148 UpDn |
| D2148 Dn F | aattTCTAGAGGAAGACTCATGCGTGCCATC | Construction of pSEVA::D2148 UpDn |
| D2148 Dn R | aattAAGCTTCATTTTCGGCGTGCCTGGTG | Construction of pSEVA::D2148 UpDn |
| D2080 sgRNA F | aattCTTAAGTCGGTGGAATAGATTTCACGGTTTTAGAGCTAGAAATAGC | Construction of pSEVA::D2080 UpDn |
| D2080 Up F | aattGGATCCGCCATCCAGCGTCGACGGC | Construction of pSEVA::D2080 UpDn |
| D2080 Up R | aattTCTAGACTGTGGTACTCCGCTTGGGC | Construction of pSEVA::D2080 UpDn |
| D2080 Dn F | aattTCTAGAGCCTTGTAGTGAGGTAAAAC | Construction of pSEVA::D2080 UpDn |
| D2080 Dn R | aattACTAGTCTACATGGAACGCATCGAGG | Construction of pSEVA::D2080 UpDn |
| D2126 sgRNA F | aattCTTAAGAACGGCATAATCTGACGTCAGTTTTAGAGCTAGAAATAGC | Construction of pSEVA::D2126 UpDn |
| D2126 Up F | aattGGATCCGGATGACACCCTCGGTAACG | Construction of pSEVA::D2126 UpDn |
| D2126 Up R | CTACAGGTCATGGTTGGTAA | Construction of pSEVA::D2126 UpDn |
| D2126 Dn F | *ACCAACCATGACCTGTA*GTCAGCCTGGATGACTGAGCA | Construction of pSEVA::D2126 UpDn |
| D2126 Dn R | aattAAGCTTCAGTTGTTCCTCAACATGGC | Construction of pSEVA::D2126 UpDn |
| D1182 sgRNA F | aattCTTAAGTTCGTCGGCCAATAGATAGAGTTTTAGAGCTAGAAATAGC | Construction of pSEVA::D1182 UpDn |
| D1182 Up F | aattGGATCCGACCTGGTGATCCTCGACCT | Construction of pSEVA::D1182 UpDn |
| D1182 Up R | TCATGGCGCCTCGCCCACG | Construction of pSEVA::D1182 UpDn |
| D1182 Dn F | *GTGGGCGAGGCGCCATGA*AGGTCAGACGCTGACCAGGC | Construction of pSEVA::D1182 UpDn |
| D1182 Dn R | aattACTAGTCACCTGGACGGTCGCGACTA | Construction of pSEVA::D1182 UpDn |
| D1780 sgRNA F | aattCTTAAGGTCGGCCAACGATTTAAGCTGTTTTAGAGCTAGAAATAG | Construction of pSEVA::D1780 UpDn |
| D1780 Up F | aattGGATCCGTCAAGCAACTGGCTGATGG | Construction of pSEVA::D1780 UpDn |
| D1780 Up R | TCACCGGATGCACTCCACAG | Construction of pSEVA::D1780 UpDn |
| D1780 Dn F | *GTGGAGTGCATCCGGTGA*TTGCGTACGCTTGGCCATC | Construction of pSEVA::D1780 UpDn |
| D1780 Dn R | aattAAGCTTGTGCTCAATCAGCTAAGGCG | Construction of pSEVA::D1780 UpDn |
| D4187 sgRNA F | aattCTTAAGCGAGGGCGTTGAGGTTAACGGTTTTAGAGCTAGAAATAG | Construction of pSEVA::D4187 UpDn |
| D4187 Up F | aattGGATCCGTCGGTGTTGCCGTGTCCAG | Construction of pSEVA::D4187 UpDn |
| D4187 Up R | AAAAGATTCCTTATCAGCTAC | Construction of pSEVA::D4187 UpDn |
| D4187 Dn F | *GCTGATAAGGAATCTTTT*TTATAAGAAACCACGGCGGG | Construction of pSEVA::D4187 UpDn |
| D4187 Dn R | aattAAGCTTGACTACAGCGCCCAGGTACA | Construction of pSEVA::D4187 UpDn |
| D5245 VF | GCTCATGGGGTGACTCCTTG | Verification of PpKT-D5245 mutant strain |
| D5245 VR | GCATTGAGCTGCTTGCTCTC | Verification of PpKT-D5245 mutant strain |
| D2148 VF | GTCTTCTCGATCAGGATGCG | Verification of PpKT-D2148 mutant strain |
| D2148 VR | CCTGCCTTCCTCGGCATAGC | Verification of PpKT-D2148 mutant strain |
| D2080 VF | CAGTACCGGCCAGGATGTTC | Verification of PpKT-D2080 mutant strain |
| D2080 VR | GGACATCCGCGAGATCCTG | Verification of PpKT-D2080 mutant strain |
| D2126 VF | CACGTTGCGTGATGTGGCC | Verification of PpKT-D2126 mutant strain |
| D2126 VR | GCAAGAAGACGAGCGCCAG | Verification of PpKT-D2126 mutant strain |
| D1182 VF | CATACCCCGCATCCTCATCG | Verification of PpKT-D1182 mutant strain |
| D1182 VR | CTGGCAACCGACGACTTCCA | Verification of PpKT-D1182 mutant strain |
| D1780 VF | GATCGTGGCGCCATCCAATC | Verification of PpKT-D1780 mutant strain |
| D1780 VR | CTGGAAATGGCCCTTGACCG | Verification of PpKT-D1780 mutant strain |
| D4187 VF | GAAGACCCACAATGGCGTGC | Verification of PpKT-D4187 mutant strain |
| D4187 VR | GACCAGTGGGTCGATGGTGG | Verification of PpKT-D4187 mutant strain |

^a^ Underlined sequences for restriction enzyme recognition sites; ^b^ Italicized sequences for overlapping between DNA sequences.

**Supplementary References**

[1] Zhang Y, Wang M, Li D, Si J, Bian X, Niu G, *et al*. Isolation, identification, and genomic analysis of a prodigiosin-producing strain of *Serratia marcescens*. Acta Microbiol Sin 2024;64:4290-4307. https://doi.org/10.13343/j.cnki.wsxb.20240303

[2] Kirill AD Barry LW. One-step inactivation of chromosomal genes in *Escherichia coli* K-12 using PCR products. Proc Natl Acad Sci USA 2000;97:6640-6645. https://doi: 10.1073/pnas.120163297

[3] Nelson KE, Weinel C, Paulsen IT, Dodson RJ, Hilbert H, Martins dos Santos VAP, *et al*. Complete genome sequence and comparative analysis of the metabolically versatile *Pseudomonas putida* KT2440. Environ Microbiol 2002;4:799-808. https://doi.org/10.1046/j.1462-2920.2002.00366.x

[4] Gust B, Challis LG, Fowler K, Kieser T Chater FK. PCR-targeted *Streptomyces* gene replacement identifies a protein domain needed for biosynthesis of the sesquiterpene soil odor geosmin. Proc Natl Acad Sci USA 2003;1541-1546. https://doi: 10.1073/pnas.0337542100

[5] Sun J, Wang Q, Jiang Y, Wen Z, Yang L, Wu J, *et al*. Genome editing and transcriptional repression in *Pseudomonas putida* KT2440 *via* the type II CRISPR system. Microb Cell Fact 2018;17. https://doi.org/10.1186/s12934-018-0887-x

[6] Li J, Mu X, Dong W, Chen Y, Kang Q, Zhao G, *et al*. A non-carboxylative route for the efficient synthesis of central metabolite malonyl-CoA and its derived products. Nat Catal 2024;7:361-374. https://doi.org/10.1038/s41929-023-01103-2
